# Synthesis and Telomeric G-Quadruplex-Stabilizing Ability of Macrocyclic Hexaoxazoles Bearing Three Side Chains

**DOI:** 10.3390/molecules24020263

**Published:** 2019-01-11

**Authors:** Yue Ma, Keisuke Iida, Shogo Sasaki, Takatsugu Hirokawa, Brahim Heddi, Anh Tuân Phan, Kazuo Nagasawa

**Affiliations:** 1 Department of Biotechnology and Life Science, Tokyo University of Agriculture and Technology, 2-24-16 Naka-cho, Koganei, Tokyo 184-8588, Japan; yue-ma@m2.tuat.ac.jp (Y.M.); s319964u@st.go.tuat.ac.jp (S.S.); 2 Department of Chemistry, Chiba University, 1-33 Yayoi, Inage, Chiba 263-8522, Japan; kiida@chiba-u.jp; 3 Transborder Medical Reserch Center, University of Tsukuba, 1-1-1 Tennodai, Tsukuba 305-8575, Japan; t-hirokawa@aist.go.jp; 4 Division of Biomedical Science, University of Tsukuba, 1-1-1 Tennodai, Tsukuba 305-8575, Japan; 5 Molecular Profiling Research Center for Drug Discovery, National Institute of Advanced Industrial Science and Technology, 2-4-7 Aomi, Koto-ward, Tokyo 135-0064, Japan; 6 Laboratoire de Biologie et Pharmacologie appliquée, CNRS UMR 8113, ENS paris-saclay 60 avenue du president Wilson, 94230 Cachan, France; brahim.heddi@ens-cachan.fr; 7 Division of Physics and Applied Physics, Nanyang Technological University, Singapore 637371, Singapore; PhanTuan@ntu.edu.sg

**Keywords:** macrocyclic oxazole, G-quadruplex, telomere, telomestatin

## Abstract

G-quadruplexes (G4s), which are structures formed in guanine-rich regions of DNA, are involved in a variety of significant biological functions, and therefore “sequence-dependent” selective G4-stabilizing agents are required as tools to investigate and modulate these functions. Here, we describe the synthesis of a new series of macrocyclic hexaoxazole-type G4 ligand (6OTD) bearing three side chains. One of these ligands, **5b**, stabilizes telomeric G4 preferentially over the G4-forming DNA sequences of *c-kit* and *K-ras*, due to the interaction of its piperazinylalkyl side chain with the groove of telomeric G4.

## 1. Introduction

G-quadruplex (G4) is one of the characteristic higher-order structures of non-B DNAs [1]. G4-forming DNA sequences have been found in telomeres, CpG islands, and gene-promoter regions [2,3,4,5,6,7], and play critical roles in many biological processes, including replication [8,9], gene transcription [10], and translation [11,12,13]. Thus, compounds that selectively stabilize G4s are promising tools for investigating those functions, as well as candidate therapeutic agents for diseases related to the G4s. Since the G4 structure consists of two or three G-quartet planes with grooves, most G4 ligands so far reported have targeted the G-quartet or the grooves [14,15]. For example, the G4 ligands TMPyP4 [16,17], PhenDC3 [18], and pyridostatin [14,19] interact with the G-quartet in G4, whereas TOxaPy [20] and distamycin A [21] recognize the groove in telomeric G4.

We have developed a series of macrocyclic hexaoxazoles, called 6OTD [22], as G4 ligands based upon the structure of the natural G4 ligand telomestatin [23,24]. Among these derivatives, we have recently shown that 4,2-L2H2-6OTD [25] and L2G2-2M2EG-6OTD [26], obtained by altering the connectivity of the oxazoles or by introducing side chains into the 6OTD core structure, interact selectively with anti-parallel and parallel-type topologies, respectively. Here, we describe the synthesis of a new series of 6OTD derivatives bearing side chains that recognize both the G-quartet and the grooves of G4. One of these ligands, **5b**, stabilizes telomeric G4 preferentially over the G4-forming DNA sequences of *c-kit* and *K-ras*, due to the interaction of a piperazinylalkyl side chain with the groove of telomeric G4 [27,28,29,30,31]. Selective stabilization of telomeric G4 would lead to the senescence and apoptosis of cancer cells through dissociation of telomeric protective proteins such as TRF2 and POT1 from telomeric DNA [32,33,34,35,36].

## 2. Results and Discussion

### 2.1. Design of 6OTD Derivatives for Targeting Telomeric G4

**Figure 1:**
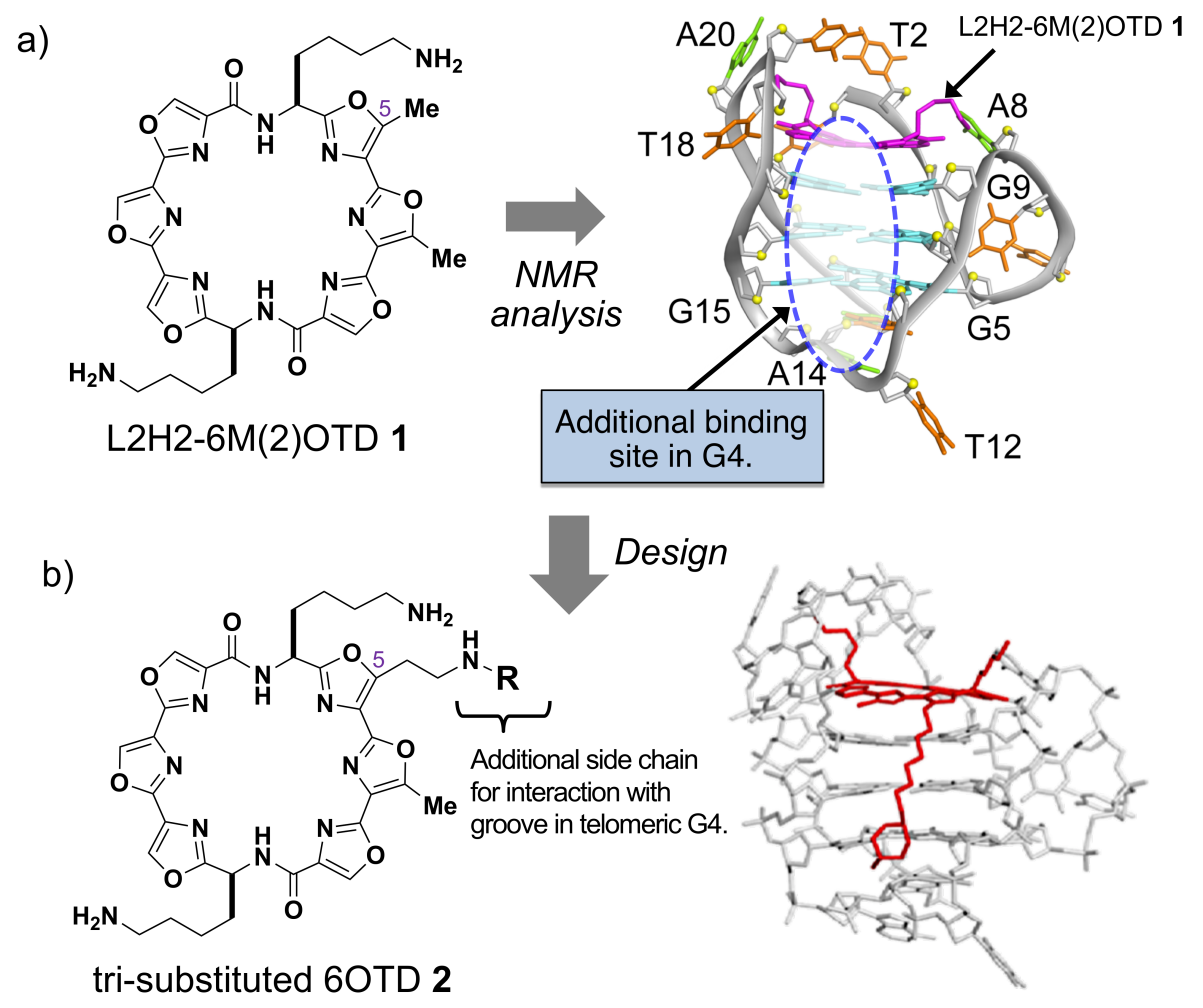
(**a**) Structure of L2H2-6M(2)OTD **1** and its interaction with telomeric G-quadruplex (G4), as elucidated by NMR analysis; (**b**) Structure of tri-substituted macrocyclic hexaoxazole (6OTD) **2**, and its possible interaction with telomeric G4.

**Figure 2:**
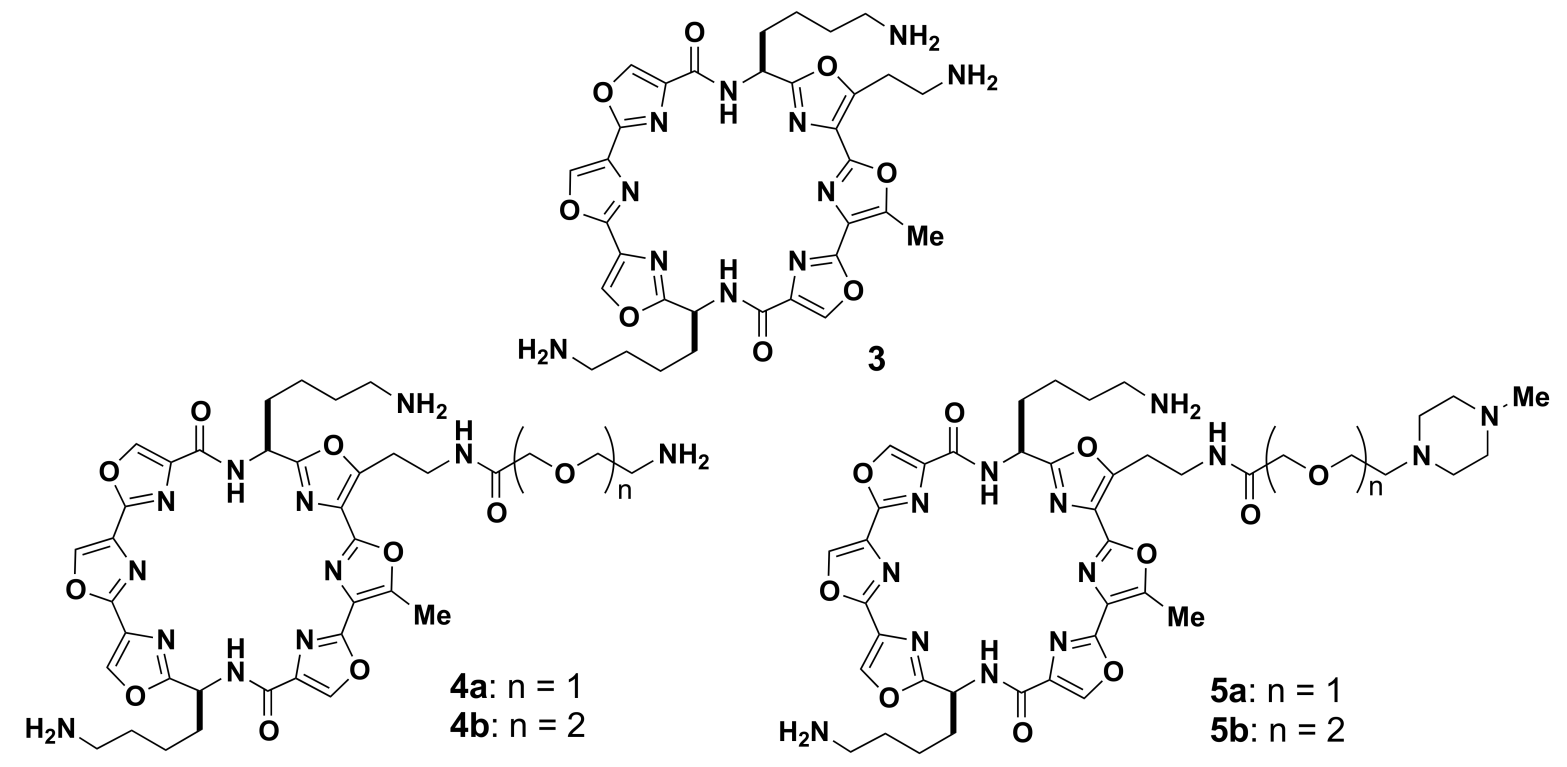
Structures of novel 6OTD analogs **3**–**5** targeting the G-quartet and the groove of telomeric G4.

G-quadruplex structures form a variety of topologies depending upon their sequences, and a promising strategy to design “sequence-dependent” selective ligands [37,38,39] is to target both the G-quartet and the G4 grooves. We previously analyzed the interaction mode of L2H2-6M(2)OTD **1** with telomeric DNA by NMR, which is shown in Figure 1a [40], and found that the core structure of the macrocyclic moiety in 6OTD **1** interacts with the G-quartet of telomeric G4 through a π-π interaction. In addition, the two aminoalkyl side chains in **1** interact with the phosphate backbone of DNA. Notably, the architecture, as shown in Figure 1a, includes an additional potential ligand-binding site, i.e., the groove circled in blue. Thus, based on the structure depicted in Figure 1b, we designed tri-substituted 6OTD **2**, which has an additional side chain at C5 of L2H2-6M(2)OTD **1**. We expected that this modification would increase the affinity for telomeric G4 and the specificity of the interaction. Specifically, we designed three types of novel tri-substituted L2H2-6OTD derivatives **3**–**5**, bearing an aminoethyl group, an aminoethoxyethyl group, and a piperazinyethoxyethyl group at C5 in **2**, respectively, and examined their specific interactions with telomeric DNA, as shown in Figure 2.

### 2.2. Synthesis of Tri-Substituted 6OTD Derivatives ***3**–**5***

**Scheme 1:**
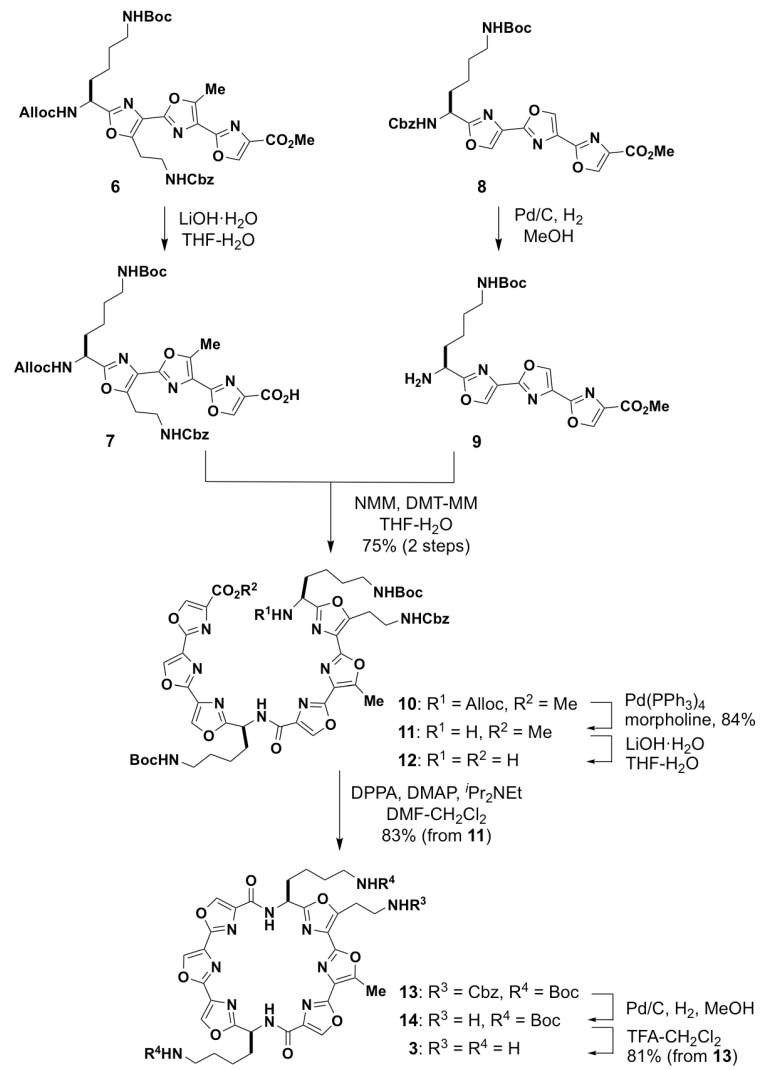
Synthesis of tri-aminoalkyl-substituted 6OTD **3.**

**Scheme 2:**
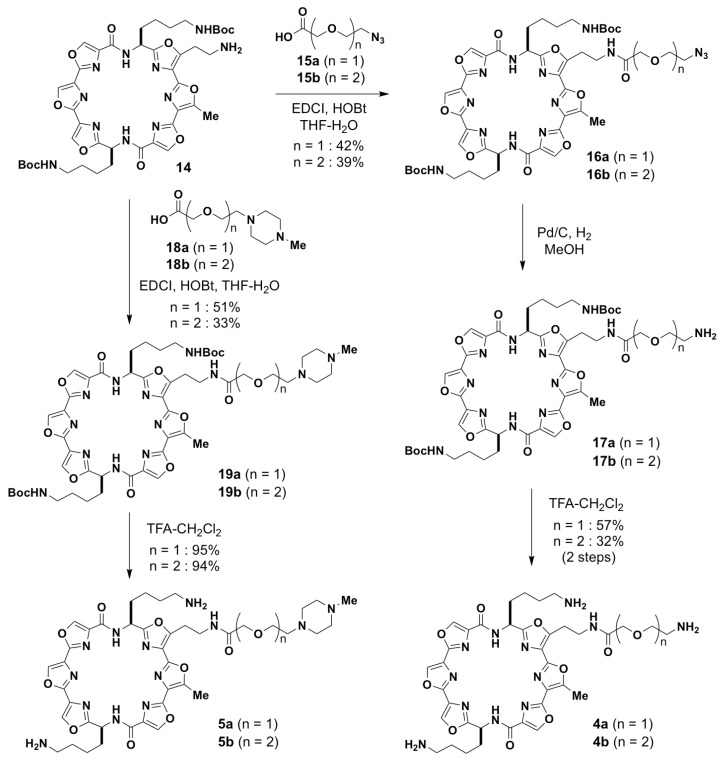
Synthesis of tri-substituted 6OTDs **4** and **5.**

We synthesized tri-substituted 6OTD derivatives **3**–**5** as summarized in Scheme 1 and Scheme 2. Firstly, the synthesis of tri-amine **3** was carried out. Briefly, the bis-Boc-protected tri-substituted 6OTD **14** was synthesized from trioxazoles **6** and **8** based upon the procedure developed by our group [41,42]. Then, the two Boc groups in **14** were deprotected with TFA to give tri-amine **3** in 81% yield from **13**. Compounds **4** and **5** were synthesized as follows: The amine **14** was reacted with carboxylic acids **15a** (n = 1) and **15b** (n = 2) bearing an azide functional group in the presence of EDCI and HOBt, and the corresponding amides **16a** and **16b** were obtained in 42% and 39% yields, respectively. After reduction of the azide group in **16** under hydrogen in the presence of Pd/C as a catalyst, the resulting amine **17** was deprotected with TFA to give tri-amines **4a** and **4b** in 94% and 90% yields from **16**, respectively. The tri-substituted L2H2-6OTD derivatives of **5a** and **5b,** bearing an *N*-methylpiperazine-substituted side chain, were synthesized similarly to **4** from amine **14** and carboxylic acids **18a** (n = 1) and **18b** (n = 2).

### 2.3. Stabilization Abilities of ***3**–**5*** for G4-Forming DNA Sequences by Fluorescence Resonance Energy Transfer (FRET) Melting Analysis

Next, the abilities of **3**–**5** to stabilize telomere, *c-kit*, and *K-ras* G4-forming DNA sequences were compared with that of L2H2-6M(2)OTD **1** by means of FRET melting assay, with results shown in Table 1 [43]. In the case of telomeric G4, telo21, the Δ*T*_m_ value was increased by ligands **3**–**5** bearing additional side chains compared with L2H2-6M(2)OTD **1**, except for with **5a**. In regards to other G4-forming DNA sequences, slight increases of Δ*T*_m_ for *c-kit* were observed with ligands **3**, **4a**, and **4b**, while significant decreases were observed with **5a** and **5b**. In the case of *K-ras*, the Δ*T*_m_ values were slightly decreased with ligands **5a** and **5b**, respectively. These results suggest that **5b** interacts selectively with telomeric G4 over the G4-forming DNA sequences of *c-kit* and *K-ras*, among the ligands and DNA sequences we examined.

### 2.4. Docking Studies of Ligands ***3***, ***4***, and ***5b*** with Telomeric G4

**Figure 3:**
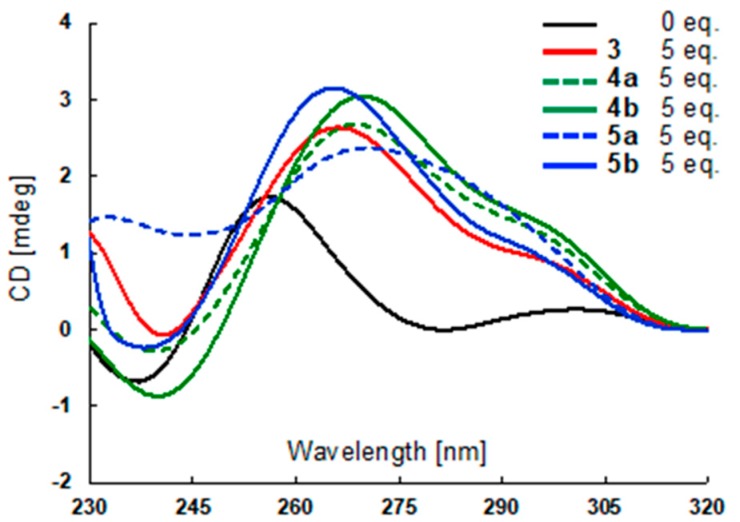
Circular Dichroism (CD) spectra of **3**, **4** and **5** in the presence of telomeric G4.

To gain insight into the differences in the stabilizing abilities of **3**, **4**, and **5b**, docking studies with telomeric G4 were carried out. Since the three topologies of telomeric DNA [44] (i.e., anti-parallel, hybrid, and parallel) are affected by ionic conditions, solvents, and ligands, we first analyzed the telomeric G4 topologies induced by ligands **3**–**5** by means of Circular Dichroism (CD) spectroscopy [45]. As shown in Figure 3, telomeric G4 was induced predominantly into the parallel form in the presence of ligands **3**, **4**, and **5b** under cation-free conditions. On the other hand, in case of ligand **5a**, the telomeric G4 was induced mainly into parallel-type topology, but did not show a single, distinctive spectrum, in contrast to the other ligands [46].

**Figure 4:**
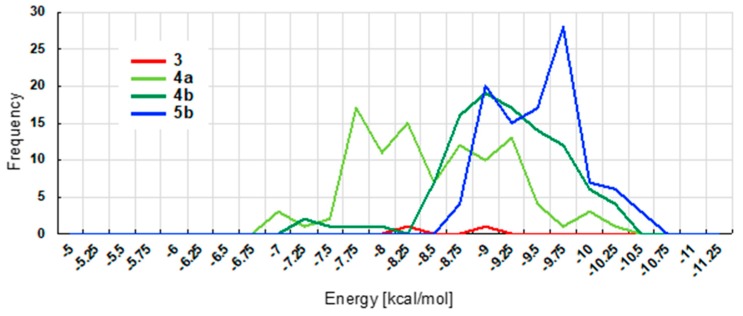
Distribution of docking energies of the top 100 poses of **3**, **4a**, **4b**, and **5b** with parallel-type telomeric G4.

With the CD analysis results in hand, docking studies were carried out between parallel-type telomeric G4 and ligands **3**, **4**, and **5b**. The docking energies between the ligands and parallel-type telomeric G4 were calculated, and the distribution of the energies for the top 100 poses is shown in Figure 4 [47]. In these calculations, **5b** mainly showed the lowest-energy docking (ca. −9 to −10.5 kcal/mol) among the ligands tested. The next lowest energy was found for **4b**, followed by **4a**, and **3**, which is consistent with the results of the FRET melting analysis, which were shown in Table 1.

**Figure 5:**
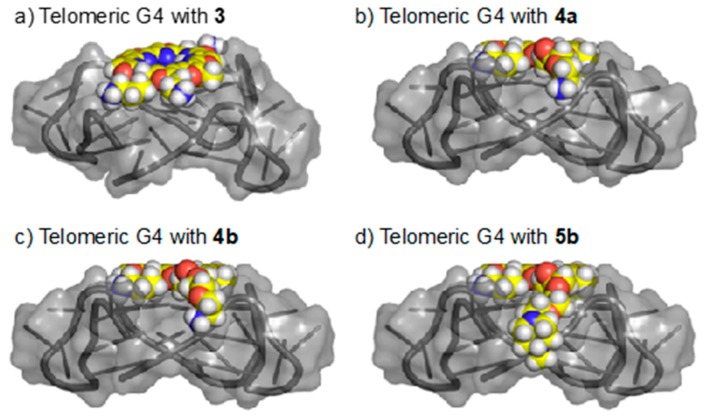
Docking models of parallel-type telomeric G4 with compounds (**a**) **3**, (**b**,**c**) **4**, and (**d**) **5b**.

Docking models with the lowest energy scores for ligands **3**, **4**, and **5b** are depicted in Figure 5. In every case, the hexaoxazole moiety in the ligands stacks with the G-quartet, and the newly introduced side chain is suggested to interact with the groove in the parallel-type telomeric G4. The two nitrogens of piperazine in the side chain in **5b** were suggested to interact efficiently with the phosphate backbone of DNA, which may contribute to **5b**’s potent stabilizing ability for telomeric DNA, compared to **3** and **4**. In the case of **5a**, the newly introduced side chain may have an inappropriate chain length for binding with the groove, resulting in a weak stabilizing ability for parallel-type telomeric G4.

## 3. Materials and Methods

### 3.1. General

Flash chromatography was performed on Silica gel 60 (spherical, particle size 0.040–0.100 mm; Kanto Co., Inc., Tokyo, Japan). Preparative-TLC (PLC) was performed using PLC Silica gel 60 F254 (0.5 mm, Merck Ltd., Darmstadt, Germany). Optical rotations were measured on a JASCO P-2200 polarimeter. ^1^H- and ^13^C-NMR spectra were recorded on JEOL JNM-AL300 (300 MHz), JEOL JNM-ECX 400 (400 MHz), and JEOL JNM-ECA 500 (500 MHz). The spectra were referenced internally according to the residual solvent signals of CDCl_3_ (^1^H-NMR, δ = 7.26 ppm; ^13^C-NMR, δ = 77.0 ppm) and DMSO-*d*_6_ (^1^H-NMR, δ = 2.50 ppm; ^13^C-NMR, δ = 39.5 ppm). Dates for ^1^H-NMR were recorded as follows: Chemical shift (δ, ppm), multiplicity (s, singlet; t, triplet; m, multiplet; br, broad), integration, and coupling constant (Hz). Dates for ^13^C-NMR were reported in terms of chemical shift (δ, ppm). Mass spectra were recorded on a JEOL JMS-T100LC spectrometer with ESI-MS mode, using MeOH as solvent.

### 3.2. Synthesis Methods

Compound **10**: To a solution of trioxazole **6** [41] (1.00 g, 1.36 mmol) in THF-H_2_O (3:1, 80 mL) was added LiOH·H_2_O (85.5 mg, 2.04 mmol) at room temperature, and stirred for 30 min. The reaction was acidified with 3 N HCl to give carboxylic acid **7** as a THF-H_2_O solution, which was used without further purification. To a solution of trioxazole **8** [42] (1.20 g, 1.40 mmol) in MeOH-THF (1:3, 80 mL) was added 10% Pd/C (120 mg), and the mixture was stirred at room temperature under a hydrogen atmosphere for 10 min. The reaction mixture was filtered through a pad of Celite^®^ (FUJIFILM Wako Pure Chemical Co., Osaka, Japan) and eluted with CHCl_3_-MeOH (9:1). The filtrates were concentrated in vacuo to give an amine **9**, which was used without further purification. To a solution of carboxylic acid **7** (THF-H_2_O) was added NMM (451 µL, 4.08 mmol), DMT-MM (1.12 g, 4.08 mmol), and amine **9**, and the mixture was stirred at room temperature. After being stirred overnight, to the reaction mixture was added H_2_O, and the organic layer was extracted with CHCl_3_, washed with 1.2 N HCl, dried over MgSO_4_, and filtered. The filtrates were concentrated in vacuo, and the residue was purified by column chromatography on silica gel (CHCl_3_:MeOH = 200:1) to give bistrioxazole **10** (1.20 g, 75%, two steps). Spectral data for **10**: ${\left[\alpha \right]}_{D}^{25}$ = −17.8 (*c* 1.1, CHCl_3_); ^1^H-NMR (400 MHz, CDCl_3_) δ 8.31–8.27 (m, 4H), 8.06 (d, *J* = 8.7 Hz, 1H), 7.26–7.21 (m, 5H), 5.90 (m, 1H), 5.47 (m, 1H), 5.33–5.20 (m, 2H), 5.03–4.94 (m, 3H), 4.66–4.57 (m, 3H), 3.95 (s, 3H), 3.73–3.62 (m, 2H), 3.45 (t, *J* = 6.4 Hz, 2H), 3.10 (br, 4H), 2.71 (s, 3H), 2.26–1.78 (m, 4H), 1.52–1.40 (m, 26H); ^13^C-NMR (100 MHz, CDCl_3_) δ 165.0, 161.1, 160.2, 156.3, 155.9, 155.8, 155.2, 151.2, 150.9, 143.8, 141.1, 139.5, 139.2, 139.1, 136.4, 136.1, 134.1, 132.4, 130.5, 129.5, 128.2, 127.8, 127.6, 126.5, 124.3, 117.7, 78.9, 78.8, 77.2, 66.5, 65.8, 54.5, 52.2, 49.0, 46.7, 40.0, 39.8, 38.7, 33.4, 32.5, 29.3, 28.2, 26.7, 22.7, 22.3, 11.6; HRMS (ESI, M + Na) calcd. for C_56_H_67_N_11_O_17_Na 1188.4614, found 1188.4628.

Compound **11**: To a solution of bistrioxazole **10** (400 mg, 340 µmol) in THF (100 mL) was added morpholine (298 µL, 3.43 mmol) and Pd(PPh_3_)_4_ (118 mg, 100 µmol) at room temperature under argon atmosphere and stirred for 10 min. The reaction mixture was concentrated in vacuo, and the residue was purified by column chromatography on silica gel (CHCl_3_:MeOH = 100:3) to give amine **11** (350 mg, 84%). Spectral data for **11**: ${\left[\alpha \right]}_{D}^{25}$ = +5.33 (*c* 1.4, CHCl_3_); ^1^H-NMR (400 MHz, CDCl_3_) δ 8.31 (s, 1H), 8.30 (s, 1H), 8.27 (s, 1H), 8.07 (d, *J* = 8.7 Hz, 1H), 7.26–7.22 (m, 7H), 5.50–5.44 (m, 1H), 5.24 (br, 1H), 5.04–4.96 (m, 2H), 4.66 (br, 1H), 4.55 (br, 1H), 4.04 (t, *J* = 6.9 Hz, 2H), 3.95 (s, 3H), 3.75–3.44 (m, 4H), 3.12–3.10 (br, 4H), 2.72 (s, 3H), 2.25–0.86 (m, 30H); ^13^C-NMR (100 MHz, CDCl_3_) δ 166.3, 165.1, 161.2, 160.3, 156.3, 155.9, 155.3, 151.1, 143.8, 141.1, 139.6, 139.2, 136.5, 136.1, 134.3, 130.6, 129.6, 128.3, 128.0, 127.7, 79.0, 78.9, 77.2, 70.5, 66.7, 52.3, 49.9, 46.8, 40.2, 40.0, 38.9, 35.4, 32.6, 29.7, 29.3, 28.3, 26.8, 22.9, 22.7, 11.8; HRMS (ESI, M + Na) calcd. for C_52_H_63_N_11_O_15_Na 1104.4403, found 1104.4370.

Compound **3**: To a solution of **13** (70.0 mg, 66.7 µmol) in MeOH (6 mL) was added 10% Pd/C (50.0 mg), and the mixture was stirred at room temperature under a hydrogen atmosphere for 10 min. The reaction mixture was filtered through a pad of Celite^®^ and eluted with CHCl_3_-MeOH (9:1). The filtrates were concentrated in vacuo to give amine **14**, which was used without further purification. The amine **14** was dissolved in CH_2_Cl_2_-TFA (10:1, 11 mL), and the resulting solution was stirred at room temperature for 10 min. The reaction mixture was concentrated in vacuo to give **3** (38.7 mg, 81%, two steps). Spectral data for **3**: ${\left[\alpha \right]}_{D}^{25}$ = +46.1 (*c* 1.1, MeOH); ^1^H-NMR (400 MHz, DMSO-*d*_6_) δ 9.14–9.12 (m, 2H), 8.92–8.90 (m, 2H), 8.33–8.27 (m, 2H), 8.15 (br, 2H), 7.75 (br, 4H), 5.45 (dt, *J* = 12.8, 5.5 Hz, 1H), 5.37 (dt, *J* = 12.8, 5.5 Hz, 1H), 3.75–3.29 (m, 4H), 2.81–2.73 (m, 7H), 2.11–1.88 (m, 4H), 1.54–1.19 (m, 8H); ^13^C-NMR (100 MHz, DMSO-*d*_6_) *δ* 164.4, 162.0, 158.9, 158.8, 158.7, 158.3, 155.7, 155.3, 155.1, 154.6, 151.9, 149.8, 142.5, 142.4, 141.8, 141.1, 136.0, 136.0, 129.7, 128.5, 125.9, 123.8, 118.4, 47.2, 47.1, 36.9, 33.3, 26.7, 23.9, 20.9, 11.5; HRMS (ESI, M + H) calcd. for C_33_H_38_N_11_O_8_ 716.2904, found 716.2918.

Compound **16a**: To a solution of **15a** (10.0 mg, 66.6 µmol) in THF-H_2_O (2:1, 3 mL) was added EDCI (38.0 mg, 200 µmol) and HOBt (27.0 mg, 200 µmol) at room temperature. After stirring for 1 h, to the reaction mixture was added amine **14** (61.0 mg, 66.6 µmol). The mixture was stirred for 24 h and quenched with 1.2 N HCl, and the organic layer was extracted with CHCl_3_, dried over MgSO_4_, and filtered. The filtrates were concentrated in vacuo, and the residue was purified by column chromatography on silica gel (CHCl_3_:MeOH = 80:1) to give **16a** (29.0 mg, 42%). Spectral data for **16a**: ${\left[\alpha \right]}_{D}^{25}$ = −14.5 (*c* 0.58, CHCl_3_); ^1^H-NMR (300 MHz, DMSO-*d*_6_) δ 9.12 (s, 1H), 9.09 (s, 1H), 8.91 (s, 1H), 8.89 (s, 1H), 8.34-8.27 (m, 2H), 7.98 (t, *J* = 6.2 Hz, 1H), 6.77 (t, *J* = 5.2 Hz, 2H), 5.42 (dt, *J* = 12.4, 7.2 Hz, 1H), 5.32 (dt, *J* = 12.4, 7.2 Hz, 1H), 3.84 (d, *J* = 15.5 Hz, 1H), 3.78 (d, *J* = 15.1 Hz, 1H), 3.61–3.27 (m, 6H), 2.84 (br, 4H), 2.75 (s, 3H), 2.04 (br, 2H), 1.90 (br, 2H), 1.33–1.03 (m, 28H); ^13^C-NMR (125 MHz, DMSO-*d*_6_) δ 169.0, 164.5, 162.5, 158.9, 158.7, 155.7, 155.5, 154.5, 154.3, 152.5, 151.0, 142.5, 142.3, 141.7, 141.0, 136.0, 135.9, 129.8, 128.4, 124.8, 124.6, 79.2, 77.2, 69.7, 69.5, 49.8, 47.4, 47.2, 36.6, 33.5, 33.4, 29.2, 28.2, 26.1, 21.1, 11.5; HRMS (ESI, M + Na) calcd. for C_47_H_58_N_14_O_14_Na 1065.4155, found 1065.4143.

Compound **4a**: To a solution of **16a** (20.0 mg, 19.2 µmol) in MeOH (4 mL) was added 10% Pd/C (20.0 mg), and the mixture was stirred at room temperature under a hydrogen atmosphere for 30 min. The reaction mixture was filtered through a pad of Celite^®^ and eluted with CHCl_3_-MeOH (9:1). The filtrates were concentrated in vacuo to give amine **17a**, which was used without further purification. The amine **17a** was dissolved in CH_2_Cl_2_-TFA (2:1, 3 mL), and the resulting solution was stirred at room temperature for 10 min. The reaction mixture was concentrated in vacuo to give **4a** (8.90 mg, 57%, two steps). Spectral data for **4a**: ${\left[\alpha \right]}_{D}^{25}$ = +87.8 (*c* 2.3, MeOH); ^1^H-NMR (500 MHz, DMSO-*d*_6_) δ 9.15 (s, 1H), 9.14 (s, 1H), 8.94 (s, 1H), 8.92 (s, 1H), 8.31 (d, *J* = 7.5 Hz, 1H), 8.28 (d, *J* = 7.5 Hz, 1H), 8.23 (t, *J* = 5.7 Hz, 1H), 7.79 (br, 6H), 5.44 (dt, *J* = 12.6, 7.5 Hz, 1H), 5.34 (dt, *J* = 12.6, 7.5 Hz, 1H), 3.84 (d, *J* = 14.9 Hz, 1H), 3.80 (d, *J* = 15.5 Hz, 1H), 3.62-3.21 (m, 4H), 3.00–2.92 (m, 2H), 2.77–2.73 (m, 7H), 2.09–1.91 (m, 4H), 1.53–1.21 (m, 10H); ^13^C-NMR (125 MHz, DMSO-*d*_6_) δ 169.0, 164.5, 158.9, 158.8, 155.7, 155.6, 155.7, 155.6, 154.6, 154.3, 152.5, 151.2, 142.6, 142.4, 141.9, 141.2, 136.0, 135.9, 129.7, 128.5, 124.7, 124.6, 69.7, 66.9, 47.2, 47.1, 38.6, 36.6, 33.5, 33.4, 26.7, 26.2, 20.9, 11.5; HRMS (ESI, M + Na) calcd. for C_37_H_44_N_12_O_10_Na 839.3201, found 839.3196.

Compound **16b**: To a solution of **15b** (15.0 mg, 77.3 µmol) in THF-H_2_O (4:1, 2.5 mL) was added EDCI (44.6 mg, 230 µmol) and HOBt (31.3 mg, 230 µmol) at room temperature. After stirring for 1 h, to the reaction mixture was added amine **14** (70.0 mg, 77.3 µmol). The mixture was stirred for 24 h and quenched with 1.2 N HCl, and the organic layer was extracted with CHCl_3_, dried over MgSO_4_, and filtered. The filtrates were concentrated in vacuo, and the residue was purified by column chromatography on silica gel (CHCl_3_:MeOH = 80:1) to give **16b** (32.7 mg, 39%). Spectral data for **16b**: ${\left[\alpha \right]}_{D}^{25}$ = −7.6 (*c* 1.7, CHCl_3_); ^1^H-NMR (500 MHz, DMSO-*d*_6_) δ 9.12 (s, 1H), 9.10 (s, 1H), 8.91 (s, 1H), 8.89 (s, 1H), 8.34–8.28 (m, 2H), 7.91 (t, *J* = 6.3 Hz, 1H), 6.78 (t, *J* = 5.7 Hz, 2H), 5.42 (dt, *J* = 12.6, 7.5 Hz, 1H), 5.32 (dt, *J* = 12.6, 7.5 Hz, 1H), 3.80 (d, *J* = 15.5 Hz, 1H), 3.75 (d, *J* = 14.9 Hz, 1H), 3.61–3.22 (m, 10H), 2.90–2.80 (m, 4H), 2.74 (s, 3H), 2.03 (br, 2H), 1.89 (br, 2H), 1.41–0.85 (m, 28H); ^13^C-NMR (125 MHz, DMSO-*d*_6_) δ 169.3, 164.6, 162.5, 158.9, 158.7, 155.7, 155.5, 154.5, 154.3, 152.5, 151.0, 142.5, 142.3, 141.7, 141.0, 136.0, 135.9, 129.8, 128.4, 124.8, 124.6, 79.2, 77.2, 70.1, 70.0, 69.3, 69.2, 50.0, 47.4, 47.2, 36.6, 33.5, 33.4, 29.2, 28.2, 29.1, 21.1, 21.0, 11.5; HRMS (ESI, M + Na) calcd. for C_49_H_62_N_14_O_15_Na 1109.4417, found 1109.4387.

Compound **4b**: To a solution of **16b** (8.00 mg, 7.35 µmol) in MeOH (1 mL) was added 10% Pd/C (10.0 mg), and the mixture was stirred at room temperature under a hydrogen atmosphere for 10 min. The reaction mixture was filtered through a pad of Celite^®^ and eluted with CHCl_3_-MeOH (9:1). The filtrates were concentrated in vacuo to give amine **17b**, which was used without further purification. The amine **17b** was dissolved in CH_2_Cl_2_-TFA (2:1, 3 mL), and the resulting solution was stirred at room temperature for 10 min. The reaction mixture was concentrated in vacuo to give **4b** (2.03 mg, 32%, 2 steps). Spectral data for **4b**: ${\left[\alpha \right]}_{D}^{25}$ = +58.2 (*c* 2.1, MeOH); ^1^H-NMR (500 MHz, DMSO-*d*_6_) δ 9.15 (s, 1H), 9.14 (s, 1H), 8.94 (s, 1H), 8.92 (s, 1H), 8.30 (d, *J* = 7.5 Hz, 1H), 8.28 (d, *J* = 8.0 Hz, 1H), 8.07 (br, 1H), 7.76 (br, 6H), 5.44 (dt, *J* = 12.6, 6.9 Hz, 1H), 5.35 (dt, *J* = 12.6, 7.5 Hz, 1H), 3.82–2.63 (m, 19H), 2.09–1.87 (m, 4H), 1.53–1.22 (m, 10H); ^13^C-NMR (125 MHz, DMSO-*d*_6_) δ 169.5, 164.5, 162.4, 159.0, 158.8, 158.4, 158.1, 155.7, 155.6, 154.6, 154.4, 152.6, 151.2, 142.6, 142.4, 141.9, 141.2, 137.4, 136.0, 135.9, 129.7, 128.9, 128.5, 128.2, 125.4, 124.7, 124.6, 69.9, 69.3, 66.7, 47.2, 47.0, 38.6, 36.6, 33.5, 33.4, 26.8, 26.1, 21.1, 21.0, 20.9, 11.6; HRMS (ESI, M + Na) calcd. for C_39_H_48_N_12_O_11_Na 883.3463, found 883.3479.

Compound **19a**: To a solution of **18a** (9.60 mg, 47.6 µmol) in THF-H_2_O (2:1, 3 mL) was added EDCI (27.4 mg, 143 µmol) and HOBt (19.3 mg, 143 µmol) at room temperature. After stirring for 1 h, to the reaction mixture was added amine 14 (43.6 mg, 47.6 µmol). The mixture was stirred for 24 h and quenched with 1.2 N HCl, and the organic layer was extracted with CHCl_3_, dried over MgSO_4_, and filtered. The filtrates were concentrated in vacuo, and the residue was purified by column chromatography on silica gel (CHCl_3_:MeOH = 10:1) to give **19a** (40.0 mg, 76%). Spectral data for **19a**: ${\left[\alpha \right]}_{D}^{25}$ = −0.25 (*c* 1.0, CHCl_3_); ^1^H-NMR (500 MHz, DMSO-*d*_6_) δ 9.12 (s, 1H), 9.09 (s, 1H), 8.91 (s, 1H), 8.90 (s, 1H), 8.32 (d, *J* = 8.0 Hz, 1H), 8.29 (d, *J* = 7.5 Hz, 1H), 8.12 (t, *J* = 6.3 Hz, 1H), 6.78 (t, *J* = 5.7 Hz, 2H), 5.42 (dt, *J* = 12.6, 7.5 Hz, 1H), 5.31 (dt, *J* = 12.6, 7.5 Hz, 1H), 3.77 (d, *J* = 15.5 Hz, 1H), 3.72 (d, *J* = 14.9 Hz, 1H), 3.62-3.24 (m, 4H), 2.84 (br, 4H), 2.74 (s, 3H), 2.36–1.88 (m, 17H), 1.33–1.03 (m, 28H); ^13^C-NMR (125 MHz, DMSO-*d*_6_) δ 169.7, 164.5, 162.5, 158.9, 158.7, 155.7, 155.5, 154.5, 154.3, 152.5, 151.0, 142.5, 142.3, 141.7, 141.0, 136.1, 136.0, 136.1, 135.9, 129.8, 128.5, 124.8, 124.7, 79.2, 77.3, 70.0, 68.5, 56.9, 54.7, 52.9, 47.4, 47.3, 45.7, 36.6, 33.4, 29.2, 28.2, 26.1, 21.1, 11.6; HRMS (ESI, M + Na) calcd. for C_52_H_69_N_13_O_14_Na 1122.4985, found 1122.4991.

Compound **5a**: To a solution of **19a** (6.70 mg, 6.01 μmol) in CH_2_Cl_2_ (2 mL) was added TFA (1 mL) at room temperature, and the mixture was stirred for 10 min. The reaction mixture was concentrated in vacuo to give **5a** (5.14 mg, 95%). Spectral data for **5a**: ${\left[\alpha \right]}_{D}^{25}$ = +85.6 (*c* 3.4, MeOH); ^1^H-NMR (500 MHz, DMSO-*d*_6_) δ 9.16 (s, 1H), 9.14 (s, 1H), 8.94 (s, 1H), 8.92 (s, 1H), 8.32 (d, *J* = 7.5 Hz, 1H), 8.30 (d, *J* = 8.0 Hz, 1H), 8.04 (t, *J* = 6.3 Hz, 1H), 7.71 (br, 4H), 5.44 (dt, *J* = 12.6, 6.9 Hz, 1H), 5.34 (dt, *J* = 12.6, 7.5 Hz, 1H), 3.81 (d, *J* = 15.5 Hz, 1H), 3.76 (d, *J* = 14.9 Hz, 1H), 3.62–3.19 (m, 15H), 2.98 (br, 2H), 2.75 (br, 7H), 2.07–1.87 (m, 4H), 1.53–1.23 (m, 10H); ^13^C-NMR (125 MHz, DMSO-*d*_6_) δ 169.1, 164.5, 162.5, 159.0, 158.8, 158.5, 155.7, 155.6, 154.6, 154.4, 152.6, 151.2, 142.6, 142.4, 141.9, 141.2, 137.4, 136.0, 135.9, 129.8, 129.0, 128.5, 128.3, 125.4, 124.8, 124.7, 117.7, 115.3, 69.5, 47.3, 47.1, 38.6, 33.5, 33.4, 26.8, 26.7, 26.0, 21.1, 21.0, 11.5; HRMS (ESI, M + H) calcd. for C_42_H_54_N_13_O_10_ 900.4117, found 900.4098.

Compound **19b**: To a solution of **18b** (9.40 mg, 38.1 µmol) in THF-H_2_O (2:1, 3 mL) was added EDCI (22.0 mg, 114 µmol) and HOBt (15.0 mg, 114 µmol) at room temperature. After stirring for 1 h, to the reaction mixture was added amine **14** (34.9 mg, 38.1 µmol). The mixture was stirred for 24 h and quenched with 1.2 N HCl, and the organic layer was extracted with CHCl_3_, dried over MgSO_4_, and filtered. The filtrates were concentrated in vacuo, and the residue was purified by column chromatography on silica gel (CHCl_3_:MeOH = 10:1) to give **19b** (14.4 mg, 33%). Spectral data for **19b**: ${\left[\alpha \right]}_{D}^{25}$ = −6.7 (*c* 0.65, CHCl_3_); ^1^H-NMR (500 MHz, DMSO-*d*_6_) δ 9.12 (s, 1H), 9.09 (s, 1H), 8.91 (s, 1H), 8.90 (s, 1H), 8.34-8.28 (m, 2H), 7.95 (t, *J* = 5.7 Hz, 1H), 6.78 (t, *J* = 5.7 Hz, 2H), 5.42 (dt, *J* = 12.6, 7.5 Hz, 1H), 5.32 (dt, *J* = 12.6, 7.5 Hz, 1H), 3.79 (d, *J* = 15.5 Hz, 1H), 3.73 (d, *J* = 15.5 Hz, 1H), 3.60–3.16 (m, 8H), 2.86–2.84 (m, 4H), 2.74 (s, 3H), 2.37–1.88 (m, 17H), 1.33–1.04 (m, 28H); ^13^C-NMR (125 MHz, DMSO-*d*_6_) δ 1 169.9, 165.1, 163.0, 162.8, 159.4, 159.2, 156.2, 156.0, 155.0, 154.8, 153.0, 151.5, 143.0, 142.8, 142.2, 141.5, 136.6, 136.4, 130.3, 129.0, 125.3, 125.1, 79.7, 77.7, 70.5, 70.4, 69.9, 68.7, 57.7, 55.2, 53.5, 47.9, 47.7, 46.3, 37.1, 36.3, 34.0, 33.9, 29.7, 28.7, 26.6, 21.6, 21.5, 12.0; HRMS (ESI, M + Na) calcd. for C_54_H_73_N_13_O_15_Na 1166.5247, found 1166.5238.

Compound **5b**: To a solution of **19b** (14.4 mg, 12.6 μmol) in CH_2_Cl_2_ (2 mL) was added TFA (1 mL) at room temperature, and the mixture was stirred for 10 min. The reaction mixture was concentrated in vacuo to give **5b** (11.2 mg, 94%). Spectral data for **5b**: ${\left[\alpha \right]}_{D}^{25}$ = +29.7 (*c* 4.4, MeOH); ^1^H-NMR (500 MHz, DMSO-*d*_6_) δ 9.15 (s, 1H), 9.14 (s, 1H), 8.94 (s, 1H), 8.92 (s, 1H), 8.31 (d, *J* = 7.5 Hz, 1H), 8.28 (d, *J* = 7.5 Hz, 1H), 8.02 (br, 1H), 7.73 (br, 4H), 5.45 (dt, *J* = 12.6, 7.5 Hz, 1H), 5.34 (dt, *J* = 12.6, 7.5 Hz, 1H), 3.80 (d, *J* = 15.5 Hz, 1H), 3.76 (d, *J* = 15.5 Hz, 1H), 3.60–3.19 (m, 19H), 2.75 (br, 9H), 2.08–1.87 (m, 4H), 1.53–1.17 (m, 10H); ^13^C-NMR (125 MHz, DMSO-*d*_6_) δ 169.4, 164.5, 162.4, 158.8, 155.7, 152.7, 151.2, 141.9, 141.2, 136.0, 128.9, 128.3, 124.8, 117.7, 115.3, 79.2, 69.9, 69.8, 69.3, 47.2, 47.1, 38.6, 33.5, 33.4, 26.8, 26.7, 26.1, 21.1, 21.0, 20.9, 11.5; HRMS (ESI, M + H) calcd. for C_44_H_58_N_13_O_11_ 944.4379, found 944.4344.

### 3.3. FRET Melting Analysis

Fluorescence resonance energy transfer (FRET) melting assay was performed with an excitation wavelength of 470–505 nm and a detection wavelength of 523–543 nm using the DNA Engine Option 2 Real-Time Cycler PCR detection system (Biorad, Hercules, CA, USA). The dual fluorescently labeled oligonucleotides were used in this protocol (Table S1). The donor fluorophore was 6-carboxyfluorescein (FAM) and the acceptor fluorophore was 6-carboxytetramethylrhodamine (TAMRA). All purified nucleotides (Sigma Genosys, Tokyo, Japan) were dissolved as stock solutions (100 μM) in MilliQ water to be used without further purification. Further dilutions of the oligonucleotides were performed with a 60 mM potassium cacodylate buffer (pH 7.4), and FRET experiments were carried out with a 0.4 μM oligonucleotide solution. Dual-labeled DNA was annealed by heating at 99 °C for 5 min, and then slowly cooled to room temperature. Ligands were prepared as DMSO stock solutions (10 mM) and diluted to 1 mM using DMSO, and then diluted to 100 μM using a 60 mM potassium cacodylate buffer (pH 7.4). Next, the annealed DNA (20 μL, 0.4 μM) and the compound solution (20 μL, 2 μM) were distributed across 96-well plates (Takara), with a total volume of 40 μL, with the labeled oligonucleotide (0.2 μM) and the compound (1.0 μM). The plates were incubated at 25 °C for 12 h. Subsequent experiments used the following temperature procedure in RT-PCR, finishing as follows: 25 °C for 20 min, and then a stepwise increase of 1 °C every minute from 25 °C until 99 °C. During the procedures, we measured the FAM fluorescence after each step. The change in the melting temperature at 1.0 μM compound concentration (*ΔT_m_* (1.0 μM)) was calculated from at least three experiments by subtraction of the blank from the averaged melting temperature of each compound.

### 3.4. CD Spectrometry

Circular Dichroism (CD) spectra were recorded on a J-720 spectropolarimeter (JASCO, Tokyo, Japan), using a quartz cell of 1 mm optical path length and an instrument scanning speed of 500 nm/min with a response time of 1 s, and over a wavelength range of 220–320 nm. The oligonucleotide telo24 used in this protocol (Table S1) was dissolved as a 1.0 mM stock solution in MilliQ water, to be used without further purification. Further dilution of the nucleotide was done with a 50 mM Tris-HCl buffer (without ions or with 50 mM KCl) from 1 mM stock solutions, to give a concentration of 10 μM. The solution was annealed by heating at 99 °C for 5 min, and then slowly cooled to room temperature, and then titrated into the oligonucleotide samples up to 5 mol equivalents using ligands, and incubated overnight. Finally, the CD spectra are representative of five averaged scans taken at 25 °C.

### 3.5. Computational Analysis

For the initial coordinates of compounds **3**, **4**, and **5**, ionization and energy minimization were performed by the OPLS3 force field in the LigPrep Script in the Maestro (Schrödinger, LLC, New York, NY, USA). These minimized structures were employed as input structures for docking simulations. The human telomeric G4 structures with parallel-type topologies (PDB ID: 1KF1 and 3UYH) were refined for docking simulations using constrained energy refinements in the OPLS-AA force field (Schrödinger LLC). Docking simulations were performed using the Glide [48,49] SP docking program (Schrödinger, LLC, New York, NY, USA).

## 4. Conclusions

In conclusion, we have newly synthesized tri-substituted 6OTD derivatives **3**–**5** by introducing aminoethyl, aminoethoxyethyl, and piperazinyethoxyethyl groups at C5 of L2H2-6M(2)OTD **1**, respectively. Among these ligands, **5b** stabilizes telomeric G4 preferentially over *c-kit* and *K-ras* G4s. Docking studies with parallel-type telomeric G4 revealed that the hexaoxazole moiety of **5b** stacks with the G-quartet of telomeric G4, and the newly introduced side chain efficiently interacts with the groove. 

## Figures and Tables

**Table 1:** Fluorescence resonance energy transfer (FRET) melting analysis of 6OTDs **3**–**5** (1.0 µM) with G4-forming DNA sequences (0.2 µM) *.

DNA	ΔT_m_ Values (°C)					
	L2H2-6M(2)OTD (1)	3	4a	4b	5a	5b
telo21	18.1 ± 0.0	23.4 ± 0.1	23.5 ± 0.2	24.4 ± 0.5	18.0 ± 0.9	26.2 ± 0.3
*c-kit*	18.3 ± 0.4	20.8 ± 0.2	19.5 ± 0.3	20.7 ± 0.1	12.9 ± 0.6	12.1 ± 0.3
*K-ras*	10.3 ± 0.4	13.0 ± 1.0	11.9 ± 0.2	13.5 ± 0.2	9.0 ± 0.2	8.2 ± 0.4
dsDNA	0.1 ± 0.0	0.0 ± 0.1	0.1 ± 0.0	0.0 ± 0.2	0.0 ± 0.1	0.0 ± 0.1

* Melting temperatures were calculated from the average and SEM of at least three experiments.

## Supplementary Materials

The following are available online.

## References

[1] Davis J.T. (2004). G-quartets 40 years later: From 5’-GMP to molecular biology and supramolecular chemistry. Angew. Chem. Int. Ed..

[2] Todd A.K., Johnston M., Neidle S. (2005). Highly prevalent putative quadruplex sequence motifs in human DNA. Nucleic Acids Res..

[3] Huppert J.L., Balasubramanian S. (2005). Prevalence of quadruplexes in the human genome. Nucleic Acids Res..

[4] Huppert J.L., Balasubramanian S. (2007). G-quadruplexes in promoters throughout the human genome. Nucleic Acids Res..

[5] Huppert J.L., Bugaut A., Kumari S., Balasubramanian S. (2008). G-quadruplexes: The beginning and end of UTRs. Nucleic Acids Res..

[6] Verma A., Yadav V.K., Basundra R., Kumar A., Chowdhury S. (2009). Evidence of genome-wide G4 DNA-mediated gene expression in human cancer cells. Nucleic Acids Res..

[7] Chambers V.S., Marsico G., Boutell J.M., Di Antonio M., Smith G.P., Balasubramanian S. (2015). High-throughput sequencing of DNA G-quadruplex structures in the human genome. Nat. Biotechnol..

[8] Paeschke K., Capra J.A., Zakian V.A. (2011). DNA replication through G-quadruplex motifs is promoted by the Saccharomyces cerevisiae Pif1 DNA helicase. Cell.

[9] Kanoh Y., Matsumoto S., Fukatsu R., Kakusho N., Kono N., Renard-Guillet C., Masuda K., Iida K., Nagasawa K., Shirahige K. (2015). Rif1 binds to G quadruplexes and suppresses replication over long distances. Nat. Struct. Mol. Biol..

[10] Siddiqui-Jain A., Grand C.L., Bearss D.J., Hurley L.H. (2002). Direct evidence for a G-quadruplex in a promoter region and its targeting with a small molecule to repress c-MYC transcription. Proc. Natl. Acad. Sci. USA.

[11] Patel D.J., Phan A.T., Kuryavyi V. (2007). Human telomere, oncogenic promoter and 5’-UTR G-quadruplexes: Diverse higher order DNA and RNA targets for cancer therapeutics. Nucleic Acids Res..

[12] Lipps H.J., Rhodes D. (2009). G-quadruplex structures: In vivo evidence and function. Trends Cell Biol..

[13] Bugaut A., Balasubramanian S. (2012). 5′-UTR RNA G-quadruplexes: Translation regulation and targeting. Nucleic Acids Res..

[14] Le D.D., Di Antonio M., Chan L.K., Balasubramanian S. (2015). G-quadruplex ligands exhibit differential G-tetrad selectivity. Chem. Commun..

[15] Ranjan N., Davis E., Xue L., Arya D.P. (2013). Dual recognition of the human telomeric G-quadruplex by a neomycin-anthraquinone conjugate. Chem. Commun..

[16] Wei C.Y., Jia G.Q., Zhou J., Han G.Y., Li C. (2009). Evidence for the binding mode of porphyrins to G-quadruplex DNA. Phys. Chem. Chem. Phys..

[17] Phan A.T., Kuryavyi V., Gaw H.Y., Patel D.J. (2005). Small-molecule interaction with a five-guanine-tract G-quadruplex structure from the human MYC promoter. Nat. Chem. Biol..

[18] Chung W.J., Heddi B., Hamon F., Teulade-Fichou M.P., Phan A.T. (2014). Solution structure of a G-quadruplex bound to the bisquinolinium compound Phen-DC3. Angew. Chem. Int. Ed..

[19] Marchand A., Granzhan A., Iida K., Tsushima Y., Ma Y., Nagasawa K., Teulade-Fichou M.P., Gabelica V. (2015). Ligand-Induced Conformational Changes with Cation Ejection upon Binding to Human Telomeric DNA G-Quadruplexes. J. Am. Chem. Soc..

[20] Hamon F., Largy E., Guedin-Beaurepaire A., Rouchon-Dagois M., Sidibe A., Monchaud D., Mergny J.L., Riou J.F., Nguyen C.H., Teulade-Fichou M.P. (2011). An Acyclic Oligoheteroaryle That Discriminates Strongly between Diverse G-Quadruplex Topologies. Angew. Chem. Int. Ed..

[21] Martino L., Virno A., Pagano B., Virgilio A., Di Micco S., Galeone A., Giancola C., Bifulco G., Mayol L., Randazzo A. (2007). Structural and thermodynamic studies of the interaction of distamycin A with the parallel quadruplex structure [d(TGGGGT)]4. J. Am. Chem. Soc..

[22] Iida K., Nagasawa K. (2013). Macrocyclic Polyoxazoles as G-Quadruplex Ligands. Chem. Rec..

[23] Kim M.Y., Vankayalapati H., Shin-Ya K., Wierzba K., Hurley L.H. (2002). Telomestatin, a potent telomerase inhibitor that interacts quite specifically with the human telomeric intramolecular g-quadruplex. J. Am. Chem. Soc..

[24] Shin-ya K., Wierzba K., Matsuo K., Ohtani T., Yamada Y., Furihata K., Hayakawa Y., Seto H. (2001). Telomestatin, a novel telomerase inhibitor from Streptomyces anulatus. J. Am. Chem. Soc..

[25] Sakuma M., Ma Y., Tsushima Y., Iida K., Hirokawa T., Nagasawa K. (2016). Design and synthesis of unsymmetric macrocyclic hexaoxazole compounds with an ability to induce distinct G-quadruplex topologies in telomeric DNA. Org. Biomol. Chem..

[26] Ma Y., Tsushima Y., Sakuma M., Sasaki S., Iida K., Okabe S., Seimiya H., Hirokawa T., Nagasawa K. (2018). Development of G-quadruplex ligands for selective induction of a parallel-type topology. Org. Biomol. Chem..

[27] Teulade-Fichou M.P., Carrasco C., Guittat L., Bailly C., Alberti P., Mergny J.L., David A., Lehn J.M., Wilson W.D. (2003). Selective recognition of G-qQuadruplex telomeric DNA by a bis(quinacridine) macrocycle. J. Am. Chem. Soc..

[28] Di Leva F.S., Zizza P., Cingolani C., D’Angelo C., Pagano B., Amato J., Salvati E., Sissi C., Pinato O., Marinelli L. (2013). Exploring the chemical space of G-quadruplex binders: Discovery of a novel chemotype targeting the human telomeric sequence. J. Med. Chem..

[29] Maji B., Kumar K., Kaulage M., Muniyappa K., Bhattacharya S. (2014). Design and synthesis of new benzimidazole-carbazole conjugates for the stabilization of human telomeric DNA, telomerase inhibition, and their selective action on cancer cells. J. Med. Chem..

[30] Lavrado J., Ohnmacht S.A., Correia I., Leitao C., Pisco S., Gunaratnam M., Moreira R., Neidle S., Santos D.J., Paulo A. (2015). Indolo [3,2-c]quinoline G-quadruplex stabilizers: A structural analysis of binding to the human telomeric G-quadruplex. Chem. Med. Chem..

[31] Abraham Punnoose J., Ma Y., Li Y., Sakuma M., Mandal S., Nagasawa K., Mao H. (2017). Adaptive and Specific Recognition of Telomeric G-Quadruplexes via Polyvalency Induced Unstacking of Binding Units. J. Am. Chem. Soc..

[32] Pennarun G., Granotier C., Gauthier L.R., Gomez D., Boussin F.D. (2005). Apoptosis related to telomere instability and cell cycle alterations in human glioma cells treated by new highly selective G-quadruplex ligands. Oncogene.

[33] Ruden M., Puri N. (2013). Novel anticancer therapeutics targeting telomerase. Cancer Treat. Rev..

[34] Xu Y. (2011). Chemistry in human telomere biology: Structure, function and targeting of telomere DNA/RNA. Chem. Soc. Rev..

[35] Balasubramanian S., Neidle S. (2009). G-quadruplex nucleic acids as therapeutic targets. Curr. Opin. Chem. Biol..

[36] Hansel-Hertsch R., Di Antonio M., Balasubramanian S. (2017). DNA G-quadruplexes in the human genome: Detection, functions and therapeutic potential. Nat. Rev. Mol. Cell Biol..

[37] Chen S.B., Hu M.H., Liu G.C., Wang J., Ou T.M., Gu L.Q., Huang Z.S., Tan J.H. (2016). Visualization of NRAS RNA G-Quadruplex Structures in Cells with an Engineered Fluorogenic Hybridization Probe. J. Am. Chem. Soc..

[38] Hu M.H., Chen S.B., Wang B., Ou T.M., Gu L.Q., Tan J.H., Huang Z.S. (2017). Specific targeting of telomeric multimeric G-quadruplexes by a new triaryl-substituted imidazole. Nucleic Acids Res..

[39] Hu M.H., Wang Y.Q., Yu Z.Y., Hu L.N., Ou T.M., Chen S.B., Huang Z.S., Tan J.H. (2018). Discovery of a New Four-Leaf Clover-Like Ligand as a Potent c-MYC Transcription Inhibitor Specifically Targeting the Promoter G-Quadruplex. J. Med. Chem..

[40] Chung W.J., Heddi B., Tera M., Iida K., Nagasawa K., Phan A.T. (2013). Solution structure of an intramolecular (3 + 1) human telomeric G-quadruplex bound to a telomestatin derivative. J. Am. Chem. Soc..

[41] Iida K., Majima S., Ohtake T., Tera M., Shin-ya K., Nagasawa K. (2012). Design and synthesis of g-quadruplex ligands bearing macrocyclic hexaoxazoles with four-way side chains. Heterocycles.

[42] Tera M., Ishizuka H., Takagi M., Suganuma M., Shin-ya K., Nagasawa K. (2008). Macrocyclic hexaoxazoles as sequence- and mode-selective G-quadruplex binders. Angew. Chem. Int. Ed..

[43] De Cian A., Guittat L., Kaiser M., Sacca B., Amrane S., Bourdoncle A., Alberti P., Teulade-Fichou M.P., Lacroix L., Mergny J.L. (2007). Fluorescence-based melting assays for studying quadruplex ligands. Methods.

[44] Ou T.M., Lu Y.J., Tan J.H., Huang Z.S., Wong K.Y., Gu L.Q. (2008). G-quadruplexes: Targets in anticancer drug design. ChemMedChem.

[45] Vorlickova M., Kejnovska I., Sagi J., Renciuk D., Bednarova K., Motlova J., Kypr J. (2012). Circular dichroism and guanine quadruplexes. Methods.

[B46-molecules-24-00263] 46.The sterically hindered side chains introduced in the compounds **4** and **5** might cause the topology change from hybrid into parallel structure by interfering with the loops.

[B47-molecules-24-00263] 47.To evaluate the docking accuracy of our protocol, we carried out the re-docking study using the known x-ray structure of G-quadruplex-ligand complex (PDB ID: 3UYH). In the re-docking experiments, we obtained near native poses (RMSD < 3 Å) in top 100 candidates, however, correlation between the docking scores and the RMSD plots were low. To avoid the choices of the false positive poses from the best-rank only, we decided to consider 100 poses together with the docking scores. See Figure S2.

[48] Friesner R.A., Banks J.L., Murphy R.B., Halgren T.A., Klicic J.J., Mainz D.T., Repasky M.P., Knoll E.H., Shelley M., Perry J.K. (2004). Glide: A new approach for rapid, accurate docking and scoring. 1. Method and assessment of docking accuracy. J. Med. Chem..

[49] Halgren T.A. (2009). Identifying and characterizing binding sites and assessing druggability. J. Chem. Inf. Model..

