# Supplementary material for: Synthesis and Telomeric G-Quadruplex-Stabilizing Ability of Macrocyclic Hexaoxazoles Bearing Three Side Chains
Source: Molecules. 2019 Jan 11;24(2):263. doi: 10.3390/molecules24020263 (PMC6358838; doi:10.3390/molecules24020263)
Supplement: Supplementary file 1 [file molecules-24-00263-s001.pdf]

Supporting information

Synthesis of macrocyclic hexaoxazoles with tri-substituted side chains and their stabilization ability of telomeric G-quadruplex

Yue Ma [1], Keisuke Iida [2], Shogo Sasaki [1], Takatsugu Hirokawa [3,4,5], Brahim Heddi [6], Anh-Tuân Phan [6], Kazuo Nagasawa [1,*]

1. Department of Biotechnology and Life Science, Tokyo University of Agriculture and Technology, 2-24-16 Naka-cho, Koganei, Tokyo 184-8588 (Japan)
2. Department of Chemistry, Chiba University, 1-33 Yayoi, Inage, Chiba 263-8522 (Japan)
3. Transborder Medical Research center, University of Tsukuba, 1-1-1 Tennodai, Tsukuba, 305-8575 (Japan)
4. Division of Biomedical Science, University of Tsukuba, 1-1-1 Tennodai, Tsukuba, 305-8575 (Japan)
5. Molecular Profiling Research Center for Drug Discovery, National Institute of Advanced Industrial Science and Technology, 2-4-7 Aomi, Koto-ward, Tokyo 135-0064 (Japan)
6. Division of Physics and Applied Physics, Nanyang Technological University, Singapore 637371 (Singapore)

*E-mail: knaga@cc.tuat.ac.jp

Table of Contents:

1. Supplementary Tables (S1, S2) S2
2. Supplementary Figures (S1, S2) S4
3. ^{1}H and ^{13}C NMR spectra for **10**, **11**, **13**, **3**, **16a**, **4a**, **16b**, **4b**, **19a**, **5a**, **19b**, **5b**. S5

1. Supplementary Tables

Table S1. Sequences of oligonucleotides used in this paper

Oligonucleotide	Sequence
Flu-telo21	5'-FAM-d[GGG TTA GGG TTA GGG TTA GGG]-TAMRA-3'
Flu-*c-kit*	5'-FAM-d[GGG AGG GCG CTG GGA GGA GGG]-TAMRA-3'
Flu-*K-ras*	5'-FAM-d[AGG GCG GTG TGG GAA GAG GGA AGA GGG GGA GG]-TAMRA-3'
Flu-dsDNA	5'-FAM-d[TAT AGC TAT ATT TTT TTA TAG CTA TA]-TAMRA-3'
telo24	5'-d[TTA GGG TTA GGG TTA GGG TTA GGG]- 3'

Table S2. Energy profiles of docking study of top 100 poses for compounds **3**, **4a**, **4b**, **5a**, and **5b** with parallel-type telomeric G4.

	3	4a	4b	5a	5b		3	4a	4b	5a	5b
**1**	-9.055	-10.328	-10.329	-10.434	-10.612	**51**		-8.501	-9.293	-9.061	-9.587
**2**	-8.48	-10.242	-10.287	-10.277	-10.594	**52**		-8.46	-9.28	-9.061	-9.577
**3**		-10.111	-10.27	-10.229	-10.544	**53**		-8.457	-9.25	-9.042	-9.563
**4**		-10.037	-10.253	-10.161	-10.492	**54**		-8.448	-9.249	-9.042	-9.562
**5**		-9.754	-10.242	-10.039	-10.441	**55**		-8.423	-9.249	-9.036	-9.556
**6**		-9.681	-10.221	-9.982	-10.396	**56**		-8.394	-9.242	-9.03	-9.551
**7**		-9.596	-10.205	-9.831	-10.378	**57**		-8.392	-9.236	-9.023	-9.551
**8**		-9.553	-10.184	-9.808	-10.356	**58**		-8.377	-9.229	-9.013	-9.55
**9**		-9.521	-10.128	-9.753	-10.344	**59**		-8.356	-9.203	-9.007	-9.513
**10**		-9.439	-10.089	-9.721	-10.202	**60**		-8.343	-9.196	-8.994	-9.51
**11**		-9.438	-9.994	-9.715	-10.188	**61**		-8.331	-9.188	-8.988	-9.503
**12**		-9.43	-9.978	-9.693	-10.038	**62**		-8.308	-9.183	-8.974	-9.47
**13**		-9.429	-9.939	-9.667	-10.035	**63**		-8.306	-9.171	-8.973	-9.451
**14**		-9.32	-9.938	-9.642	-10.032	**64**		-8.286	-9.169	-8.97	-9.447
**15**		-9.308	-9.874	-9.636	-10.03	**65**		-8.26	-9.148	-8.964	-9.437
**16**		-9.307	-9.872	-9.629	-10.002	**66**		-8.257	-9.098	-8.958	-9.412
**17**		-9.282	-9.822	-9.609	-9.97	**67**		-8.224	-9.091	-8.941	-9.412
**18**		-9.267	-9.806	-9.588	-9.97	**68**		-8.22	-9.075	-8.933	-9.397
**19**		-9.265	-9.789	-9.537	-9.967	**69**		-8.217	-9.037	-8.923	-9.392
**20**		-9.257	-9.78	-9.492	-9.963	**70**		-8.198	-9.004	-8.916	-9.389
**21**		-9.254	-9.759	-9.47	-9.956	**71**		-8.174	-9.003	-8.915	-9.388
**22**		-9.252	-9.754	-9.4	-9.953	**72**		-8.114	-9.002	-8.882	-9.34
**23**		-9.24	-9.693	-9.388	-9.941	**73**		-8.081	-8.981	-8.88	-9.339
**24**		-9.184	-9.684	-9.382	-9.927	**74**		-8.06	-8.976	-8.88	-9.313
**25**		-9.175	-9.635	-9.382	-9.914	**75**		-8.055	-8.966	-8.876	-9.299
**26**		-9.168	-9.63	-9.38	-9.908	**76**		-8.028	-8.963	-8.854	-9.255
**27**		-9.161	-9.619	-9.366	-9.875	**77**		-8.005	-8.957	-8.852	-9.238
**28**		-9.147	-9.615	-9.363	-9.87	**78**		-7.977	-8.954	-8.851	-9.23
**29**		-9.103	-9.609	-9.361	-9.869	**79**		-7.969	-8.942	-8.842	-9.201
**30**		-9.061	-9.603	-9.361	-9.858	**80**		-7.967	-8.925	-8.822	-9.173
**31**		-9.021	-9.595	-9.314	-9.856	**81**		-7.951	-8.915	-8.811	-9.169
**32**		-9.011	-9.554	-9.275	-9.853	**82**		-7.949	-8.911	-8.795	-9.169
**33**		-8.988	-9.554	-9.27	-9.847	**83**		-7.945	-8.892	-8.792	-9.157
**34**		-8.978	-9.543	-9.258	-9.833	**84**		-7.942	-8.871	-8.777	-9.147
**35**		-8.974	-9.531	-9.251	-9.822	**85**		-7.929	-8.854	-8.777	-9.132
**36**		-8.967	-9.52	-9.249	-9.815	**86**		-7.923	-8.837	-8.757	-9.124
**37**		-8.96	-9.492	-9.178	-9.804	**87**		-7.911	-8.813	-8.738	-9.12
**38**		-8.954	-9.49	-9.177	-9.803	**88**		-7.908	-8.781	-8.733	-9.087
**39**		-8.934	-9.458	-9.17	-9.789	**89**		-7.904	-8.741	-8.718	-9.084
**40**		-8.898	-9.454	-9.164	-9.778	**90**		-7.897	-8.731	-8.639	-9.083
**41**		-8.889	-9.454	-9.15	-9.775	**91**		-7.897	-8.714	-8.611	-9.083
**42**		-8.883	-9.446	-9.137	-9.765	**92**		-7.879	-8.702	-8.595	-9.067
**43**		-8.872	-9.439	-9.134	-9.762	**93**		-7.878	-8.668	-8.579	-9.056
**44**		-8.786	-9.417	-9.121	-9.752	**94**		-7.836	-8.654	-8.565	-9.047
**45**		-8.744	-9.389	-9.103	-9.704	**95**		-7.527	-8.646	-8.485	-9.039
**46**		-8.728	-9.387	-9.102	-9.68	**96**		-7.501	-8.006	-8.403	-9.015
**47**		-8.691	-9.366	-9.097	-9.671	**97**		-7.272	-7.916	-8.153	-8.965
**48**		-8.685	-9.358	-9.087	-9.668	**98**		-7.195	-7.712	-7.675	-8.931
**49**		-8.627	-9.329	-9.085	-9.647	**99**		-7.172	-7.457	-7.503	-8.916
**50**		-8.614	-9.319	-9.071	-9.62	**100**		-7.07	-7.43	-7.38	-8.827

2. Supplementary Figures

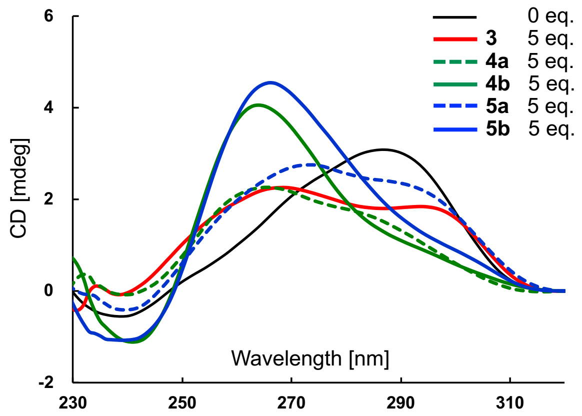

Figure S1. CD spectra of **3**, **4a**, **4b**, **5a**, and **5b** in the presence of telomeric G4 under potassium condition

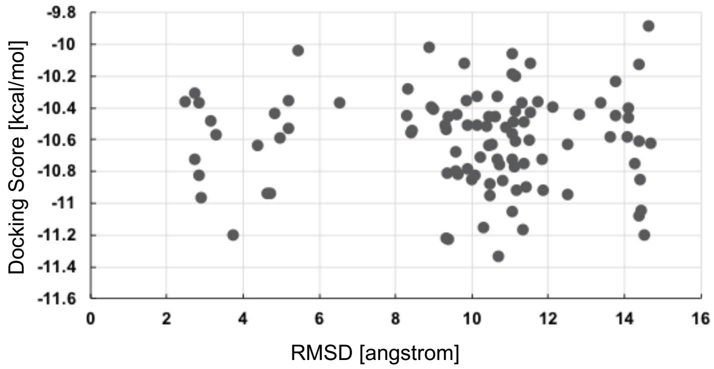

Figure S2. Correlation between the docking score and the RMSD of the top 100 poses for re-docking benchmark (PDB ID: 3UYH)

2. ^{1}H and ^{13}C NMR spectra for 10, 11, 13, 3, 16a, 4a, 16b, 4b, 19a, 5a, 19b, 5b.

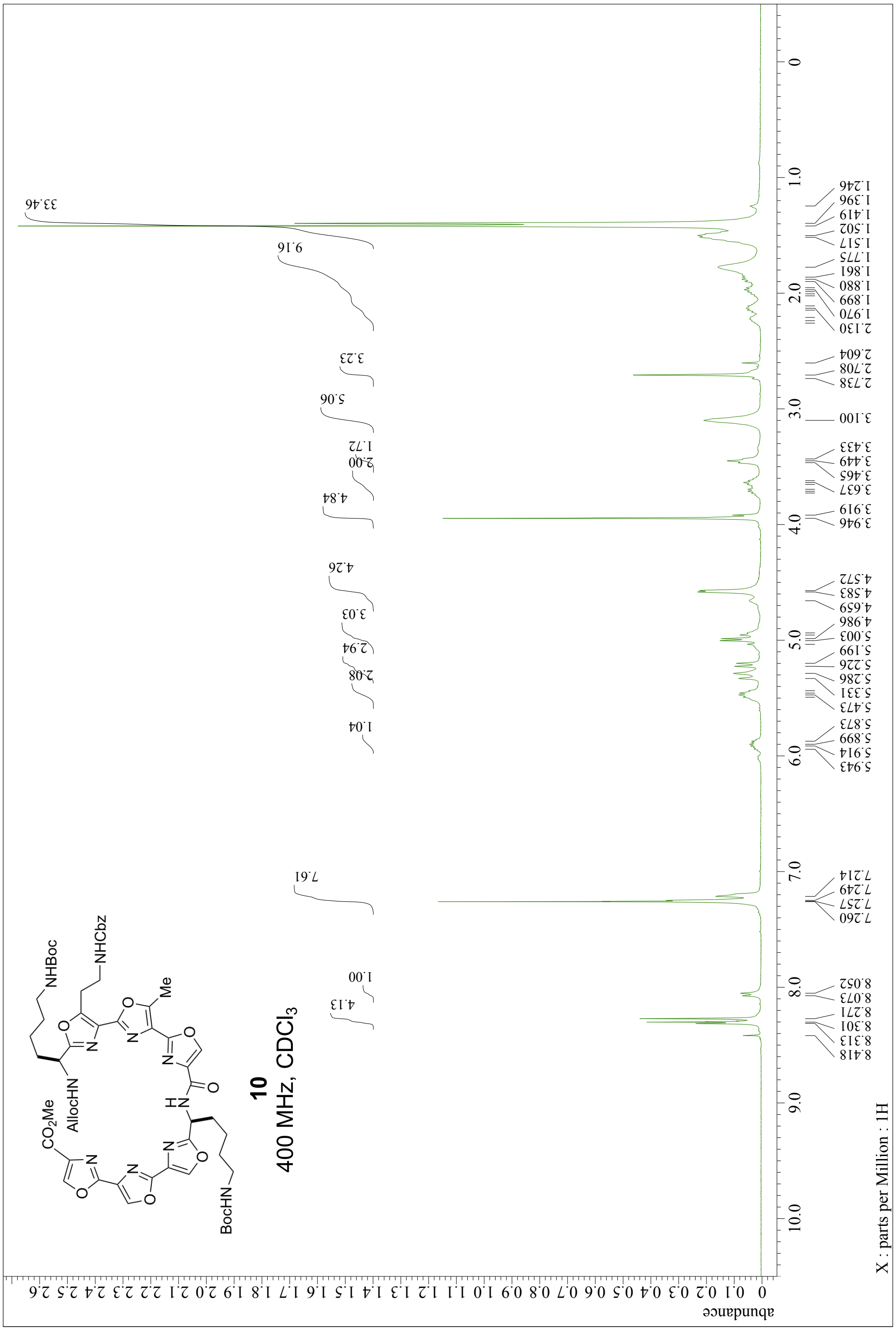

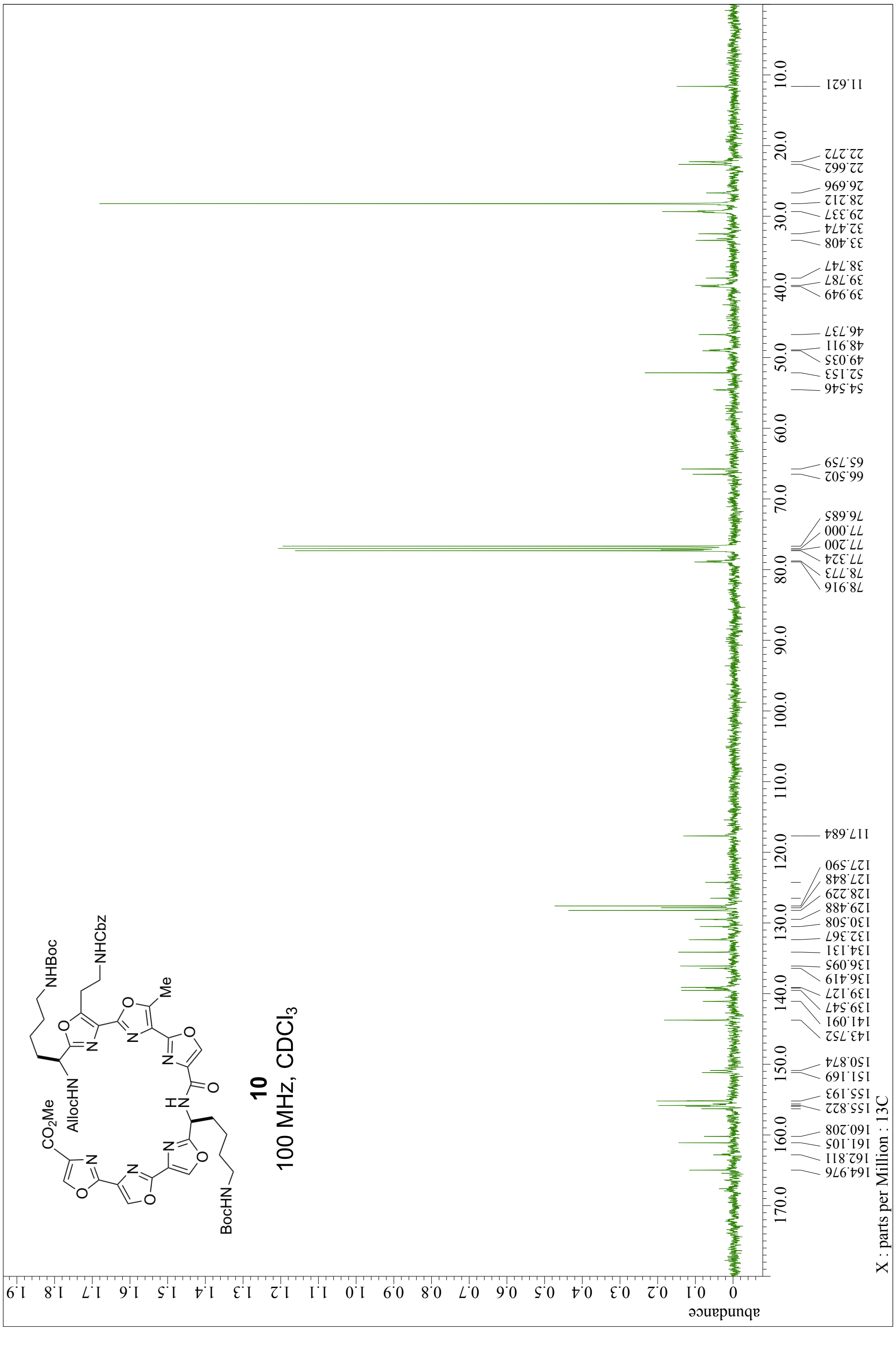
10
100 MHz, CDCl3
X : parts per Million : 13C
abundance
11.621
22.272
22.662
26.696
28.212
29.337
32.474
33.408
38.747
39.787
39.949
46.737
48.911
49.035
52.153
54.546
65.759
66.502
76.685
77.000
77.200
77.324
78.773
78.916
117.684
127.590
127.848
128.229
129.488
130.508
132.367
134.131
136.095
136.419
139.127
139.547
141.091
143.752
150.874
151.169
155.193
155.822
160.208
161.105
162.811
164.976

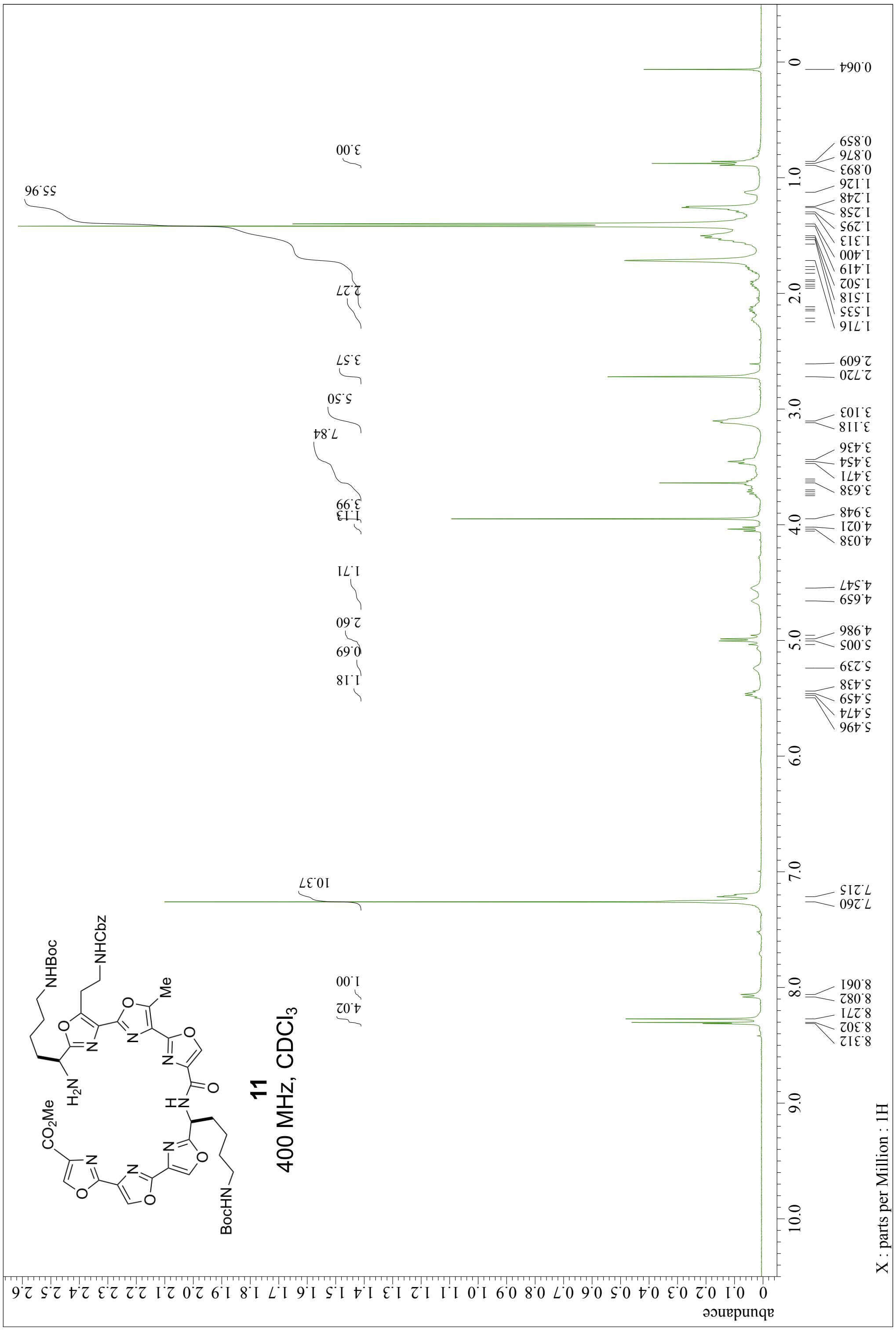

NHBoc
NHCbz
Me
CO2Me
H2N
BocHN
11
400 MHz, CDCl3
X : parts per Million : 1H
abundance

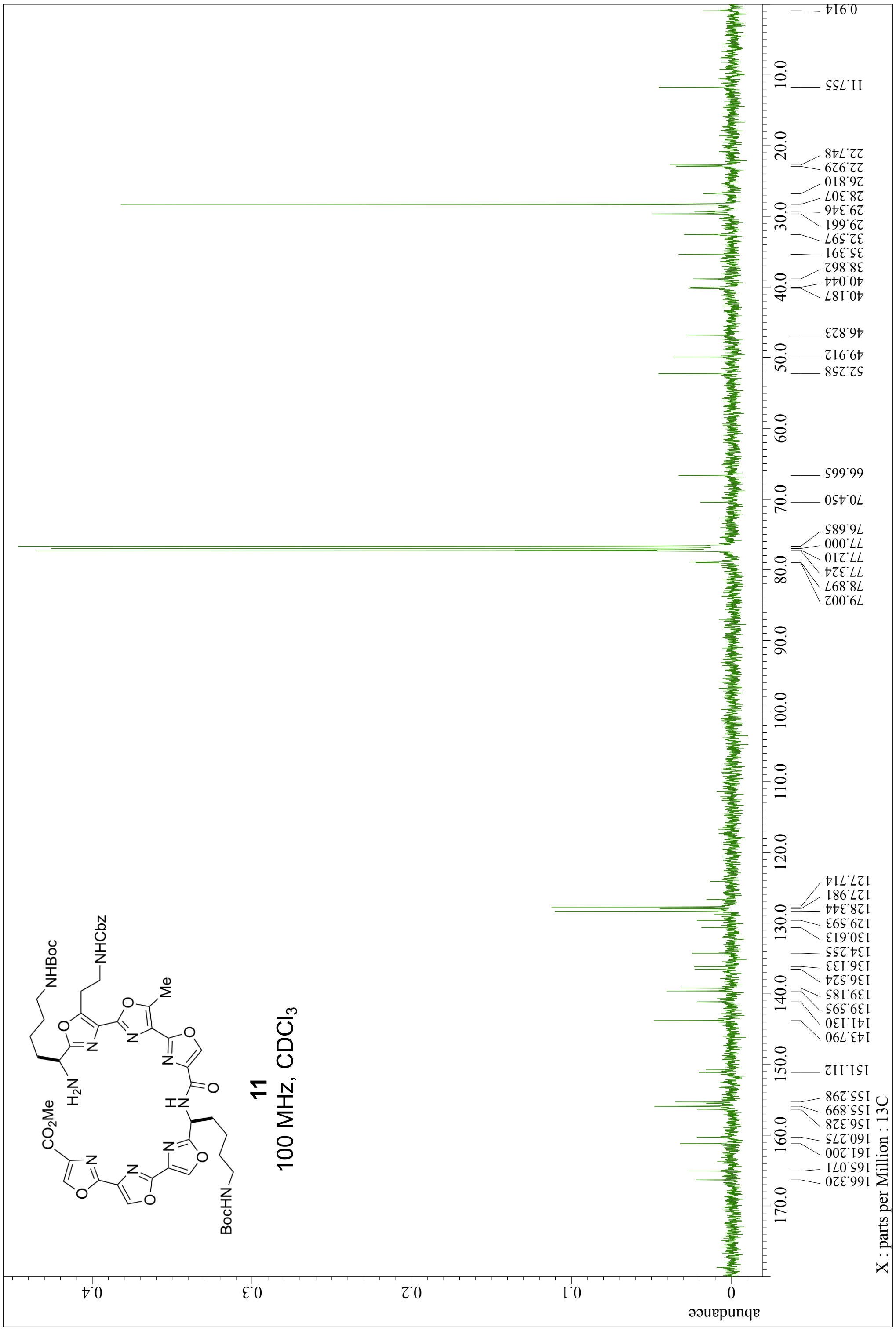
11
100 MHz, CDCl3
NHBoc
NHCbz
Me
CO2Me
H2N
BocHN
abundance
X : parts per Million : 13C
0.914
11.755
22.748
22.929
26.810
28.307
29.346
29.661
32.597
35.391
38.862
40.044
40.187
46.823
49.912
52.258
66.665
70.450
76.685
77.000
77.210
77.324
78.897
79.002
127.714
127.981
128.344
129.593
130.613
134.255
136.133
136.524
139.185
139.595
141.130
143.790
151.112
155.298
155.899
156.328
160.275
161.200
165.071
166.320

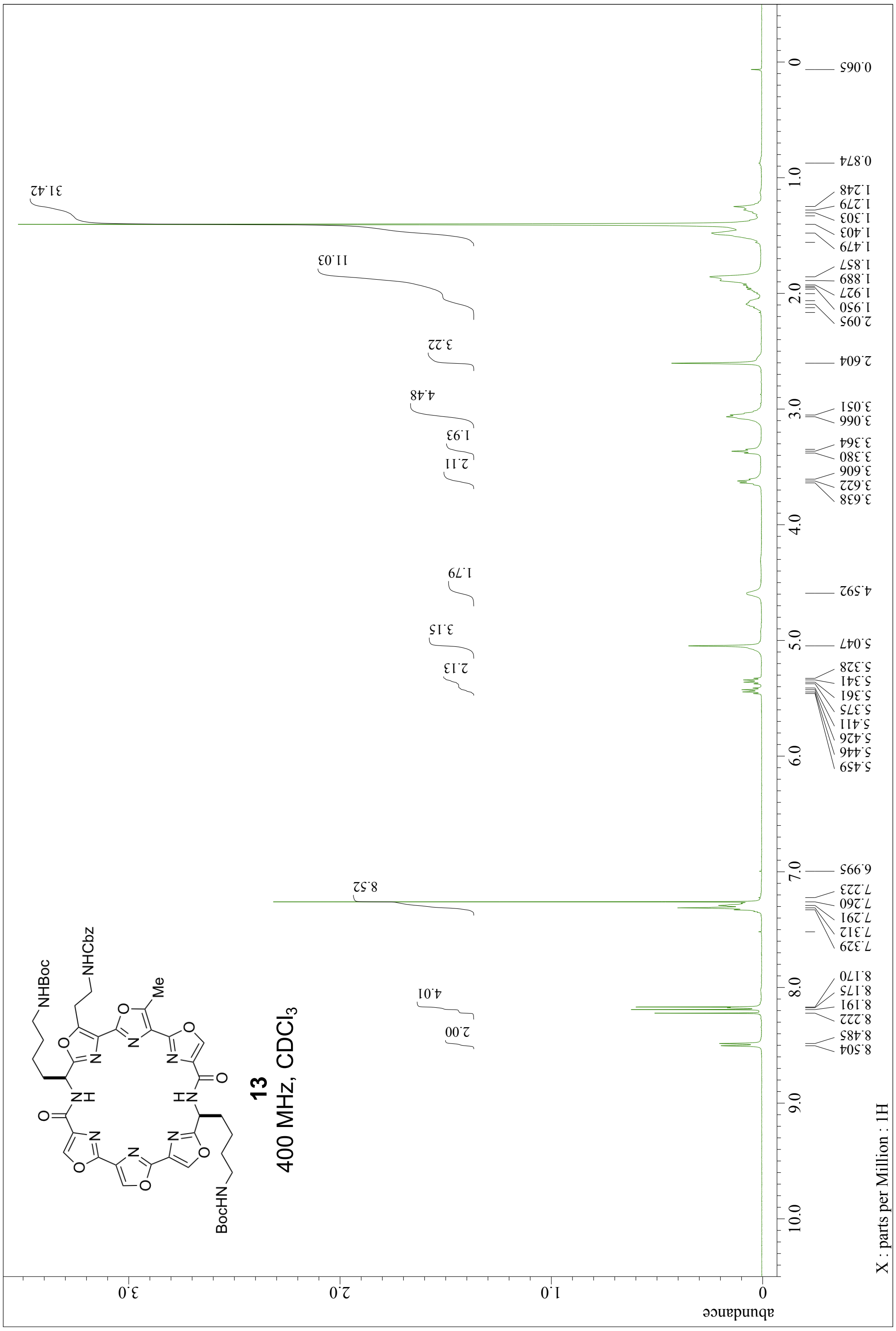
13
400 MHz, $CDCl_3$
NHBoc
NHCbz
Me
BocHN
X : parts per Million : 1H
abundance

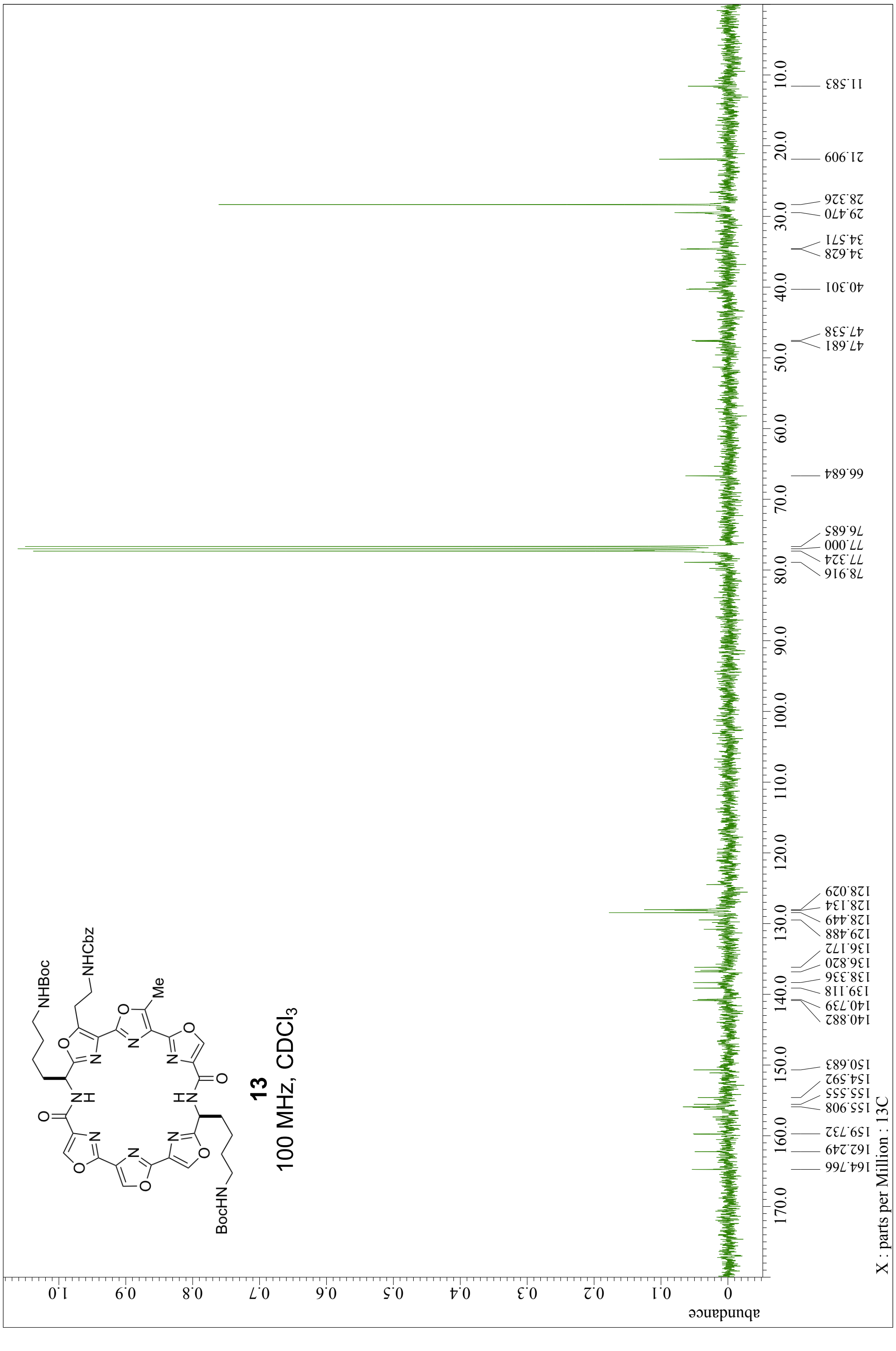
13
100 MHz, CDCl3
NHBoc
NHCbz
Me
BocHN
abundance
X : parts per Million : 13C
11.583
21.909
28.326
29.470
34.571
34.628
40.301
47.538
47.681
66.684
76.685
77.000
77.324
78.916
128.029
128.134
128.449
129.488
136.172
136.820
138.336
139.118
140.739
140.882
150.683
154.592
155.555
155.908
159.732
162.249
164.766

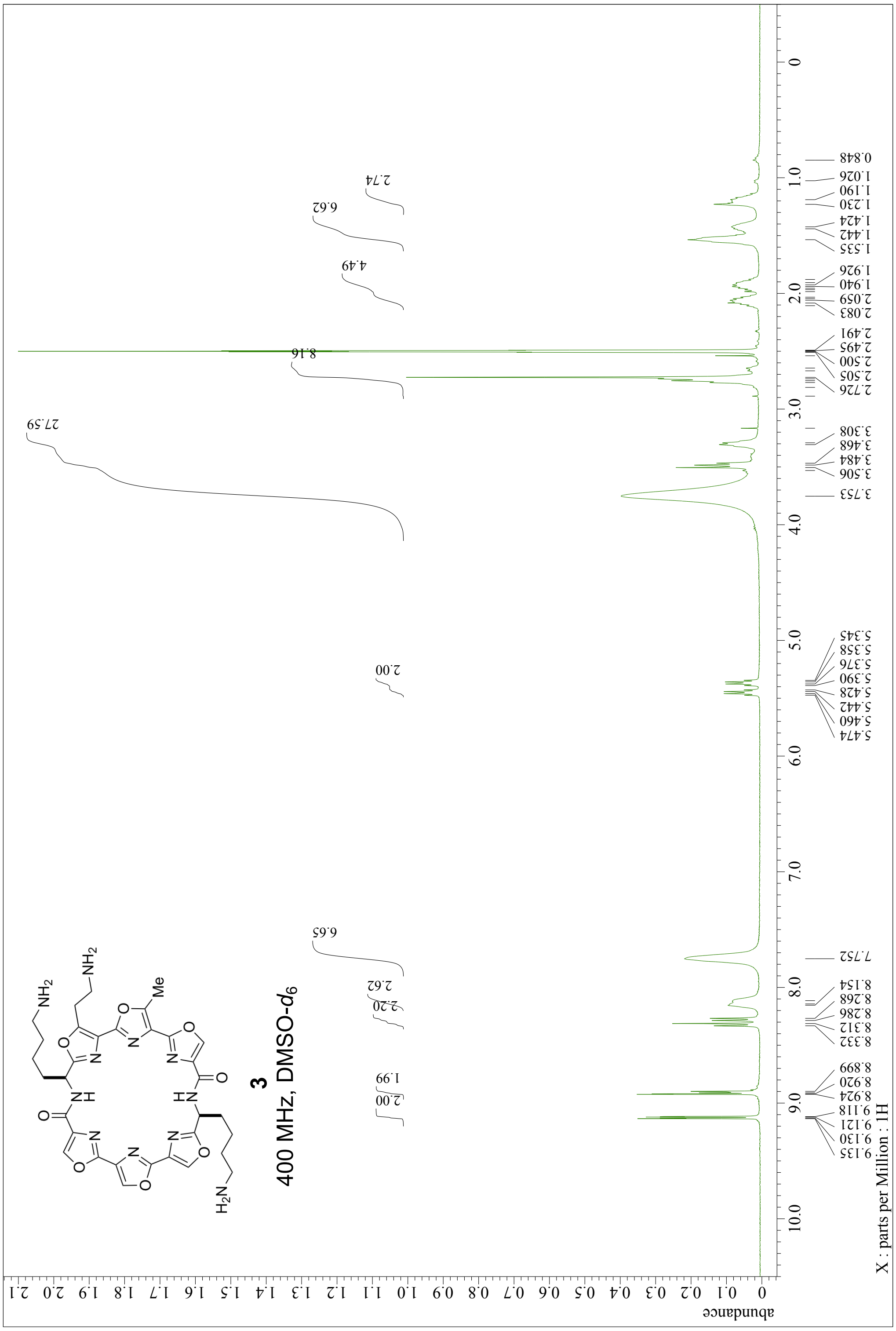
3
400 MHz, DMSO-d6
X : parts per Million : 1H
abundance
0.848
1.026
1.190
1.230
1.424
1.442
1.535
1.926
1.940
2.059
2.083
2.491
2.495
2.500
2.505
2.726
3.308
3.468
3.484
3.506
3.753
5.345
5.358
5.376
5.390
5.428
5.442
5.460
5.474
7.752
8.154
8.268
8.286
8.312
8.332
8.899
8.920
8.924
9.118
9.121
9.130
9.135
2.74
6.62
4.49
8.16
27.59
2.00
6.65
2.62
2.20
1.99
2.00

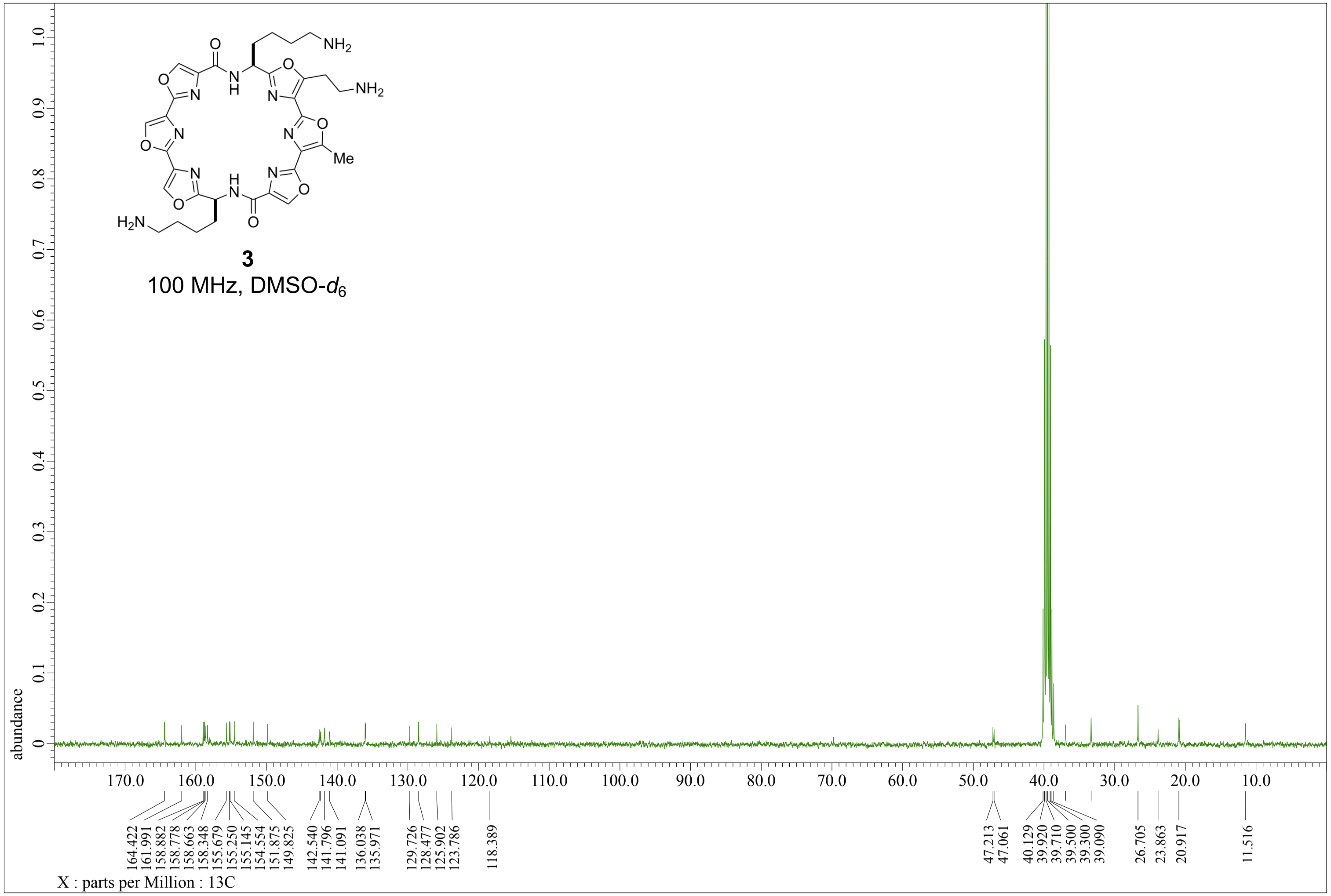
3
100 MHz, DMSO-d_6
abundance
X : parts per Million : 13C
164.422
161.991
158.882
158.778
158.663
158.348
155.679
155.250
155.145
154.554
151.875
149.825
142.540
141.796
141.091
136.038
135.971
129.726
128.477
125.902
123.786
118.389
47.213
47.061
40.129
39.920
39.710
39.500
39.300
39.090
26.705
23.863
20.917
11.516

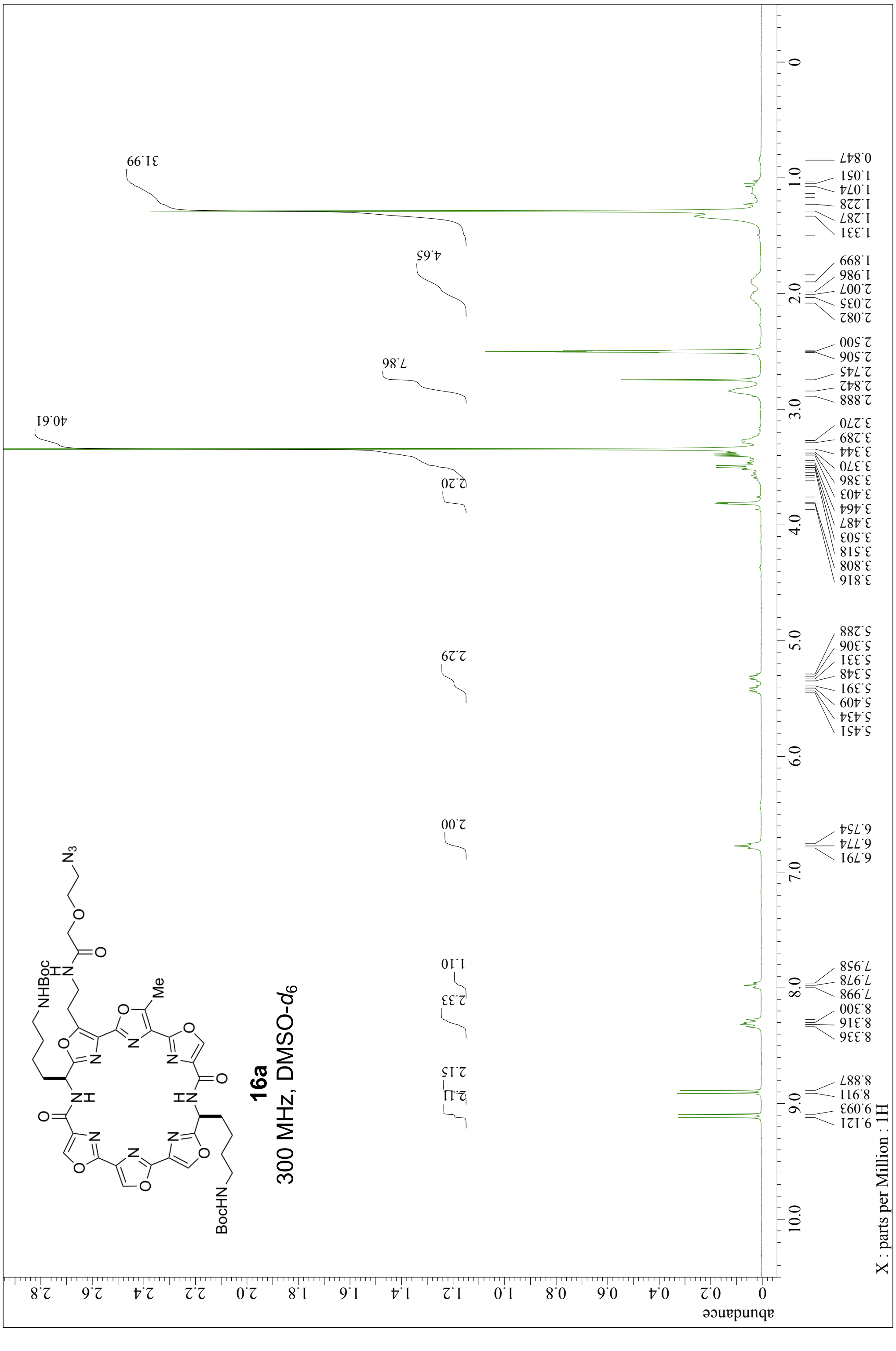
16a
300 MHz, DMSO-d_6
NHBoc
BocHN
N3
Me
X : parts per Million : 1H
abundance
0.847
1.051
1.074
1.228
1.287
1.331
1.899
1.986
2.007
2.035
2.082
2.500
2.506
2.745
2.842
2.888
3.270
3.289
3.344
3.370
3.386
3.403
3.464
3.487
3.503
3.518
3.808
3.816
5.288
5.306
5.331
5.348
5.391
5.409
5.434
5.451
6.754
6.774
6.791
7.958
7.978
7.998
8.300
8.316
8.336
8.887
8.911
9.093
9.121
31.99
4.65
7.86
40.61
2.20
2.29
2.00
1.10
2.33
2.15
2.11

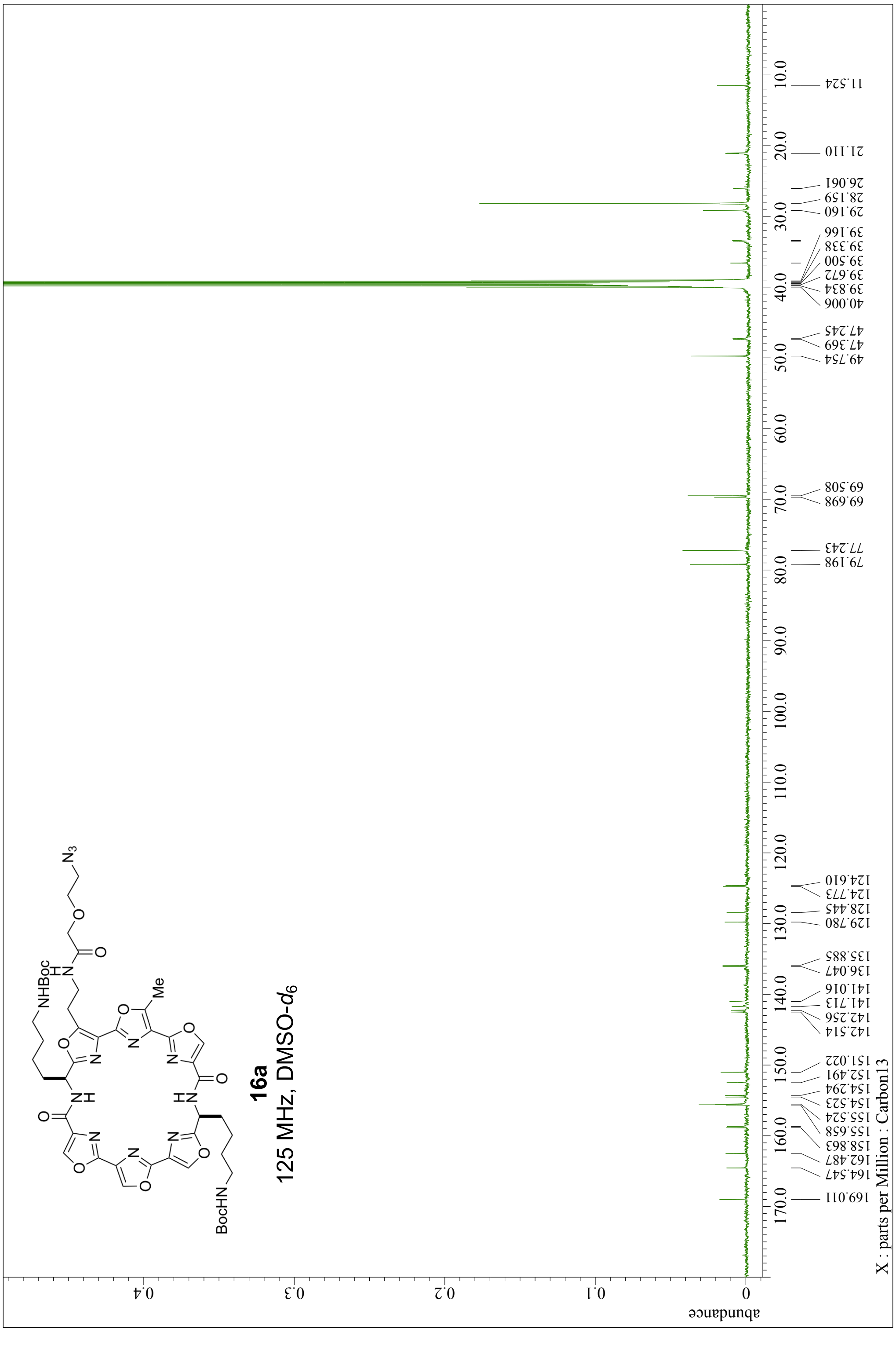
16a
125 MHz, DMSO-d_6
X : parts per Million : Carbon13
abundance
11.524
21.110
26.061
28.159
29.160
39.166
39.338
39.500
39.672
39.834
40.006
47.245
47.369
49.754
69.508
69.698
77.243
79.198
124.610
124.773
128.445
129.780
135.885
136.047
141.016
141.713
142.256
142.514
151.022
152.491
154.294
154.523
155.524
155.658
158.863
162.487
164.547
169.011

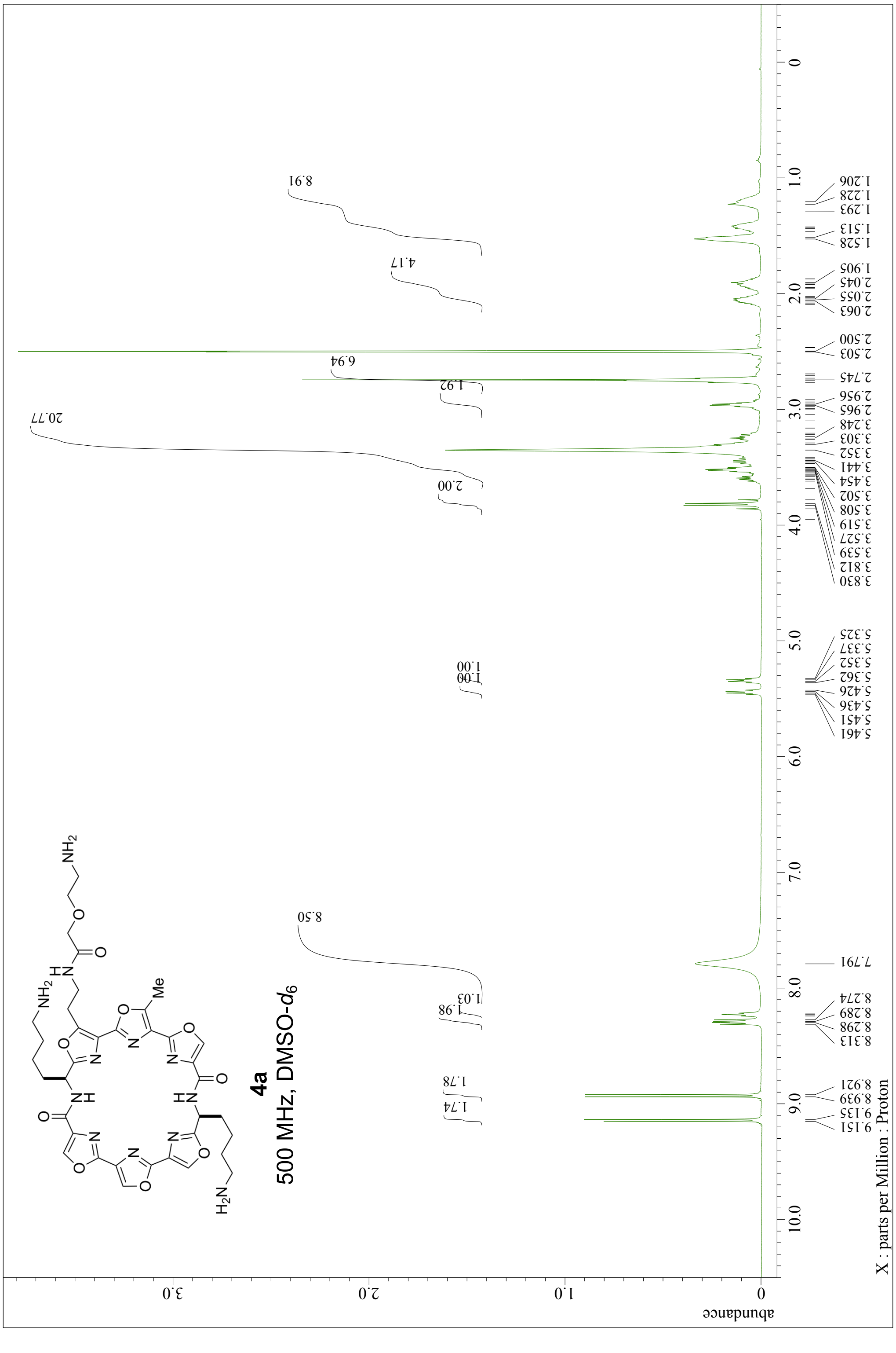
4a
500 MHz, DMSO-d_6
X : parts per Million : Proton
abundance
1.206
1.228
1.293
1.513
1.528
1.905
2.045
2.055
2.063
2.500
2.503
2.745
2.956
2.965
3.248
3.303
3.352
3.441
3.454
3.502
3.508
3.519
3.527
3.539
3.812
3.830
5.325
5.337
5.352
5.362
5.426
5.436
5.451
5.461
7.791
8.274
8.289
8.298
8.313
8.921
8.939
9.135
9.151
8.91
4.17
6.94
1.92
20.77
2.00
1.00
1.00
8.50
1.03
1.98
1.78
1.74

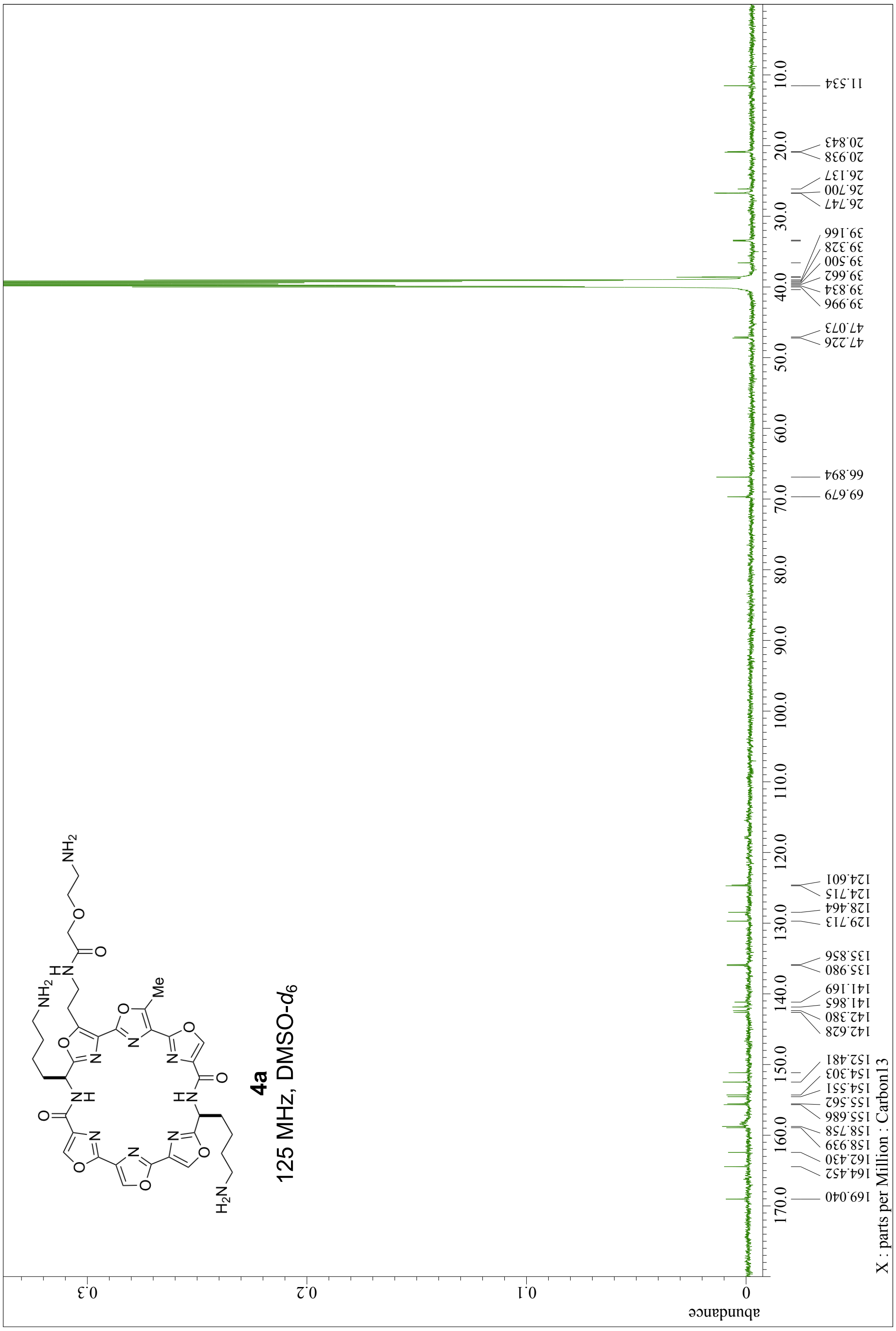
4a
125 MHz, DMSO-d_6
Me
NH_2
H_2N
11.534
20.843
20.938
26.137
26.700
26.747
39.166
39.328
39.500
39.662
39.834
39.996
47.073
47.226
66.894
69.679
124.601
124.715
128.464
129.713
135.856
135.980
141.169
141.865
142.380
142.628
152.481
154.303
154.551
155.562
155.686
158.758
158.939
162.430
164.452
169.040
X : parts per Million : Carbon13
abundance

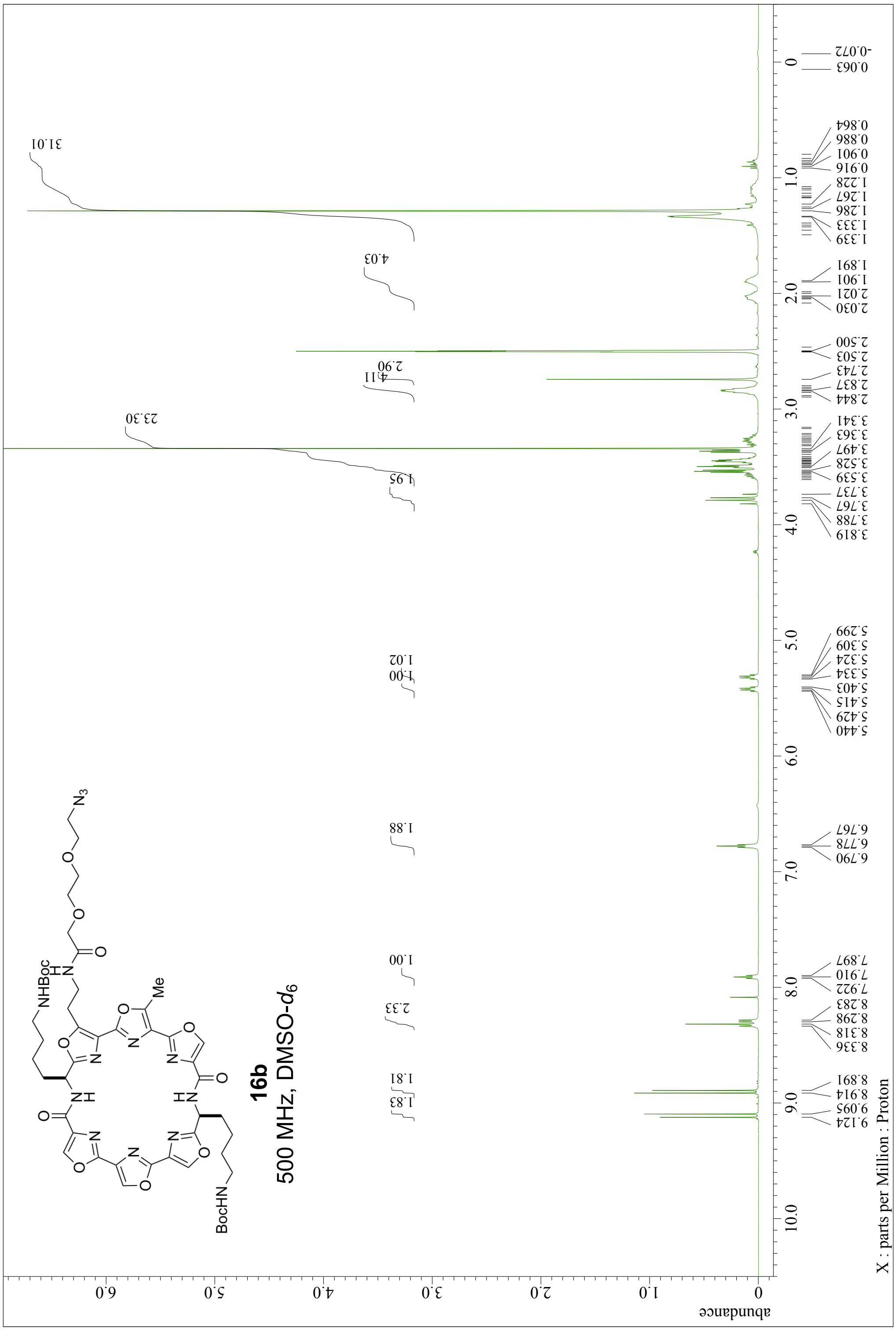
16b
500 MHz, DMSO-d_6
X : parts per Million : Proton
abundance

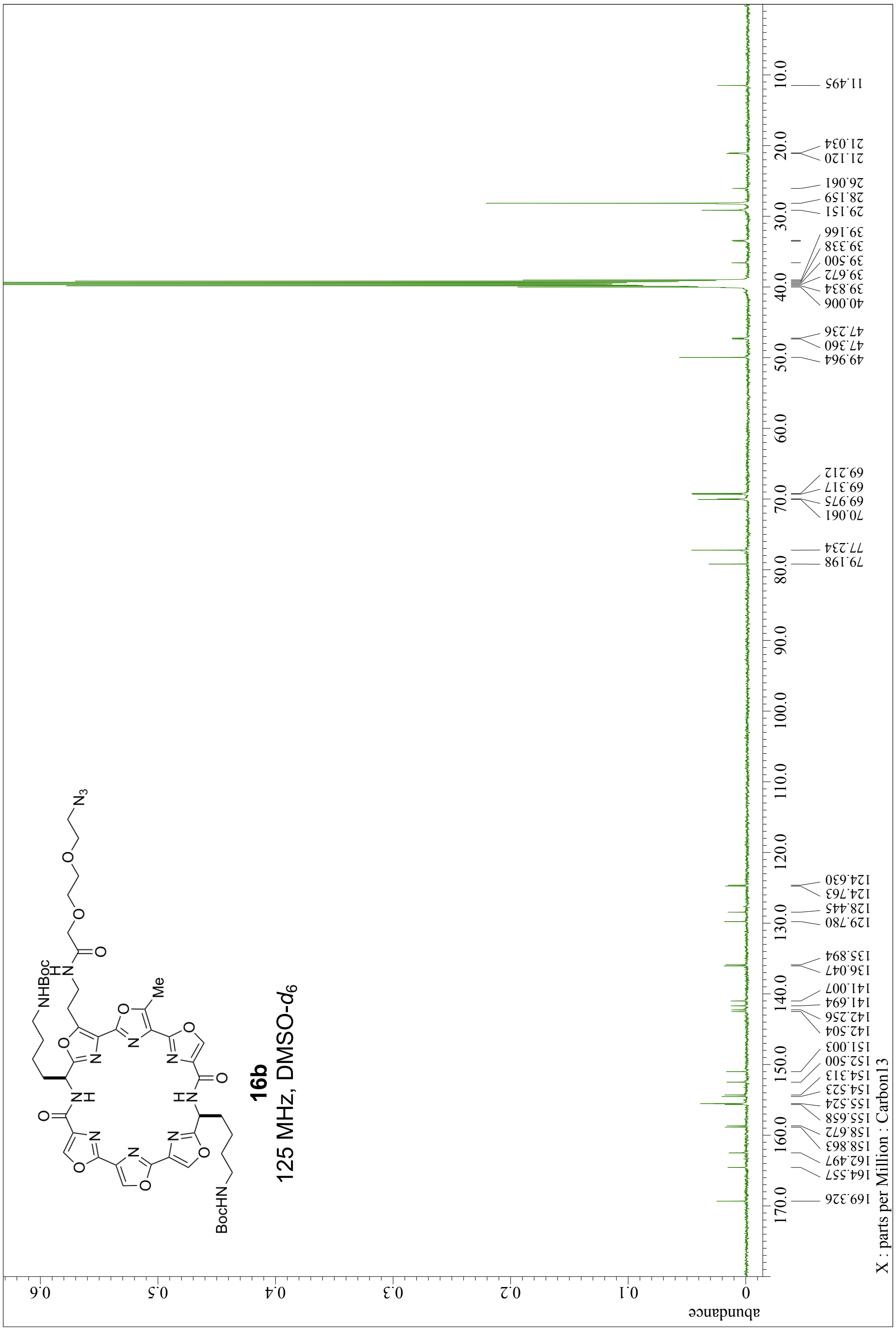
16b
125 MHz, DMSO-d_6
NHBoc
BocHN
Me
N_3
X : parts per Million : Carbon13
abundance
11.495
21.034
21.120
26.061
28.159
29.151
39.166
39.338
39.500
39.672
39.834
40.006
47.236
47.360
49.964
69.212
69.317
69.975
70.061
77.234
79.198
124.630
124.763
128.445
129.780
135.894
136.047
141.007
141.694
142.256
142.504
151.003
152.500
154.313
154.523
155.524
155.658
158.672
158.863
162.497
164.557
169.326

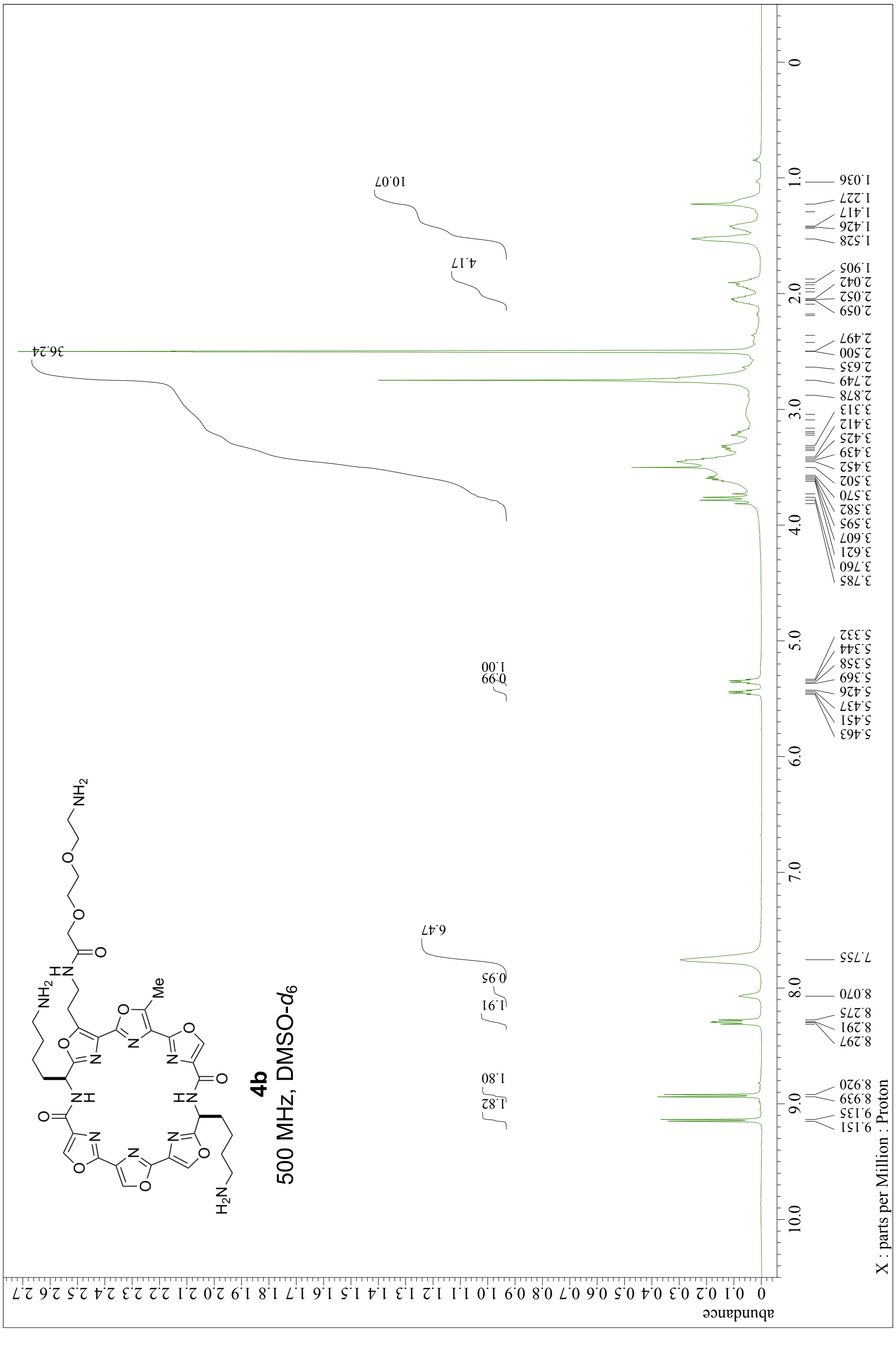

4b
500 MHz, DMSO-d_6
X : parts per Million : Proton
abundance

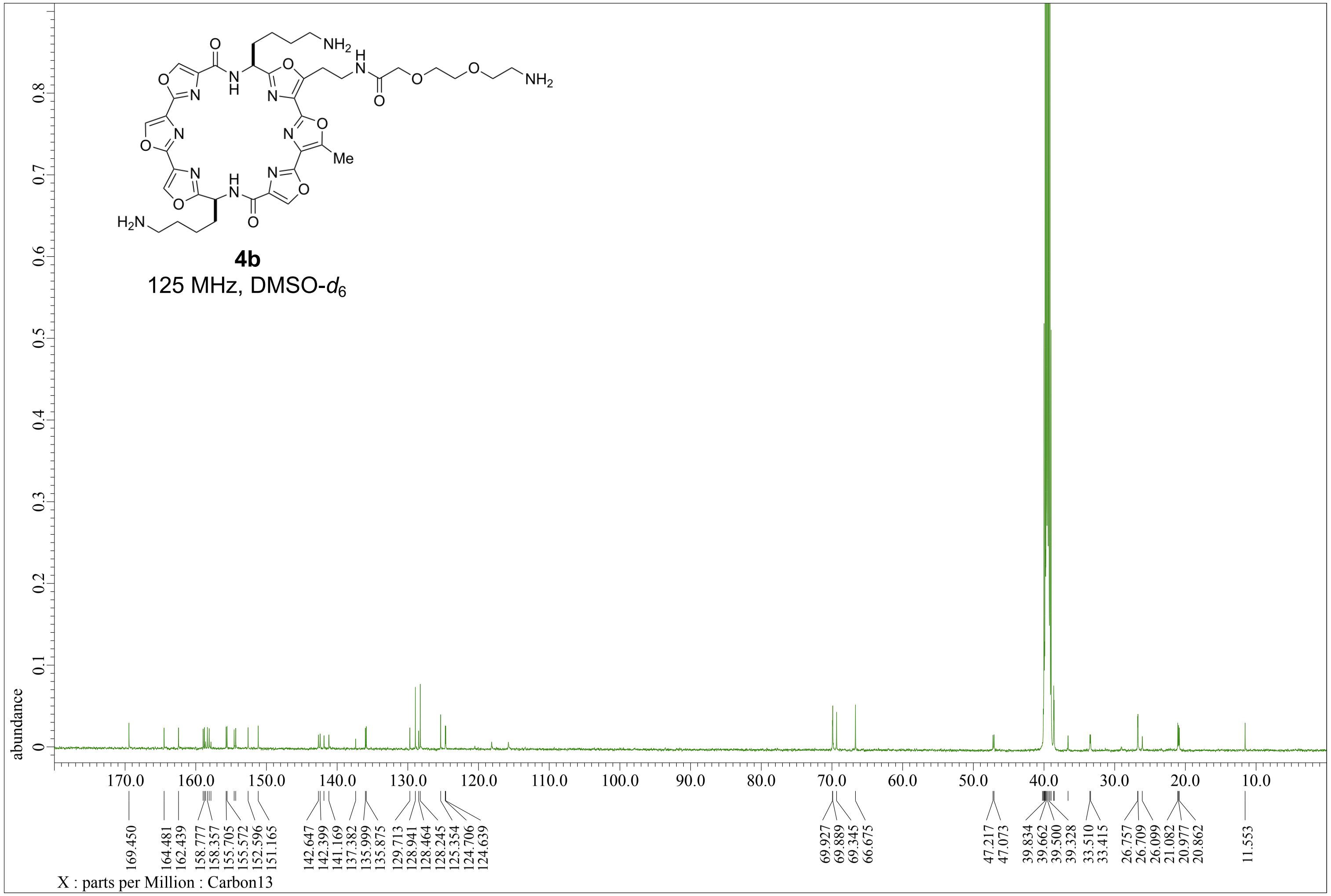
4b
125 MHz, DMSO-d6
abundance
0
0.1
0.2
0.3
0.4
0.5
0.6
0.7
0.8
170.0
160.0
150.0
140.0
130.0
120.0
110.0
100.0
90.0
80.0
70.0
60.0
50.0
40.0
30.0
20.0
10.0
169.450
164.481
162.439
158.777
158.357
155.705
155.572
152.596
151.165
142.647
142.399
141.169
137.382
135.999
135.875
129.713
128.941
128.464
128.245
125.354
124.706
124.639
69.927
69.889
69.345
66.675
47.217
47.073
39.834
39.662
39.500
39.328
33.510
33.415
26.757
26.709
26.099
21.082
20.977
20.862
11.553
X : parts per Million : Carbon13

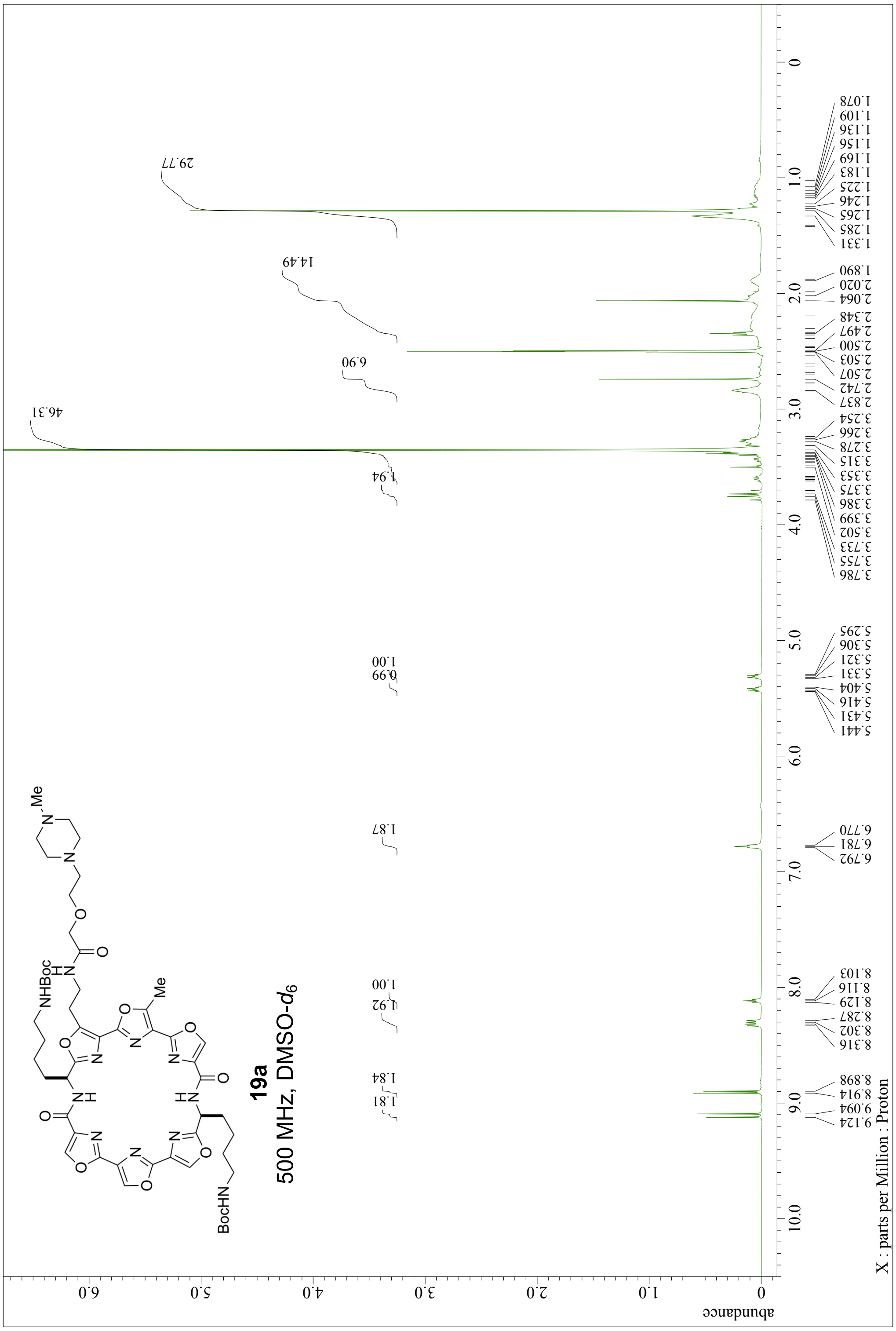
19a
500 MHz, DMSO-d6
X : parts per Million : Proton
abundance

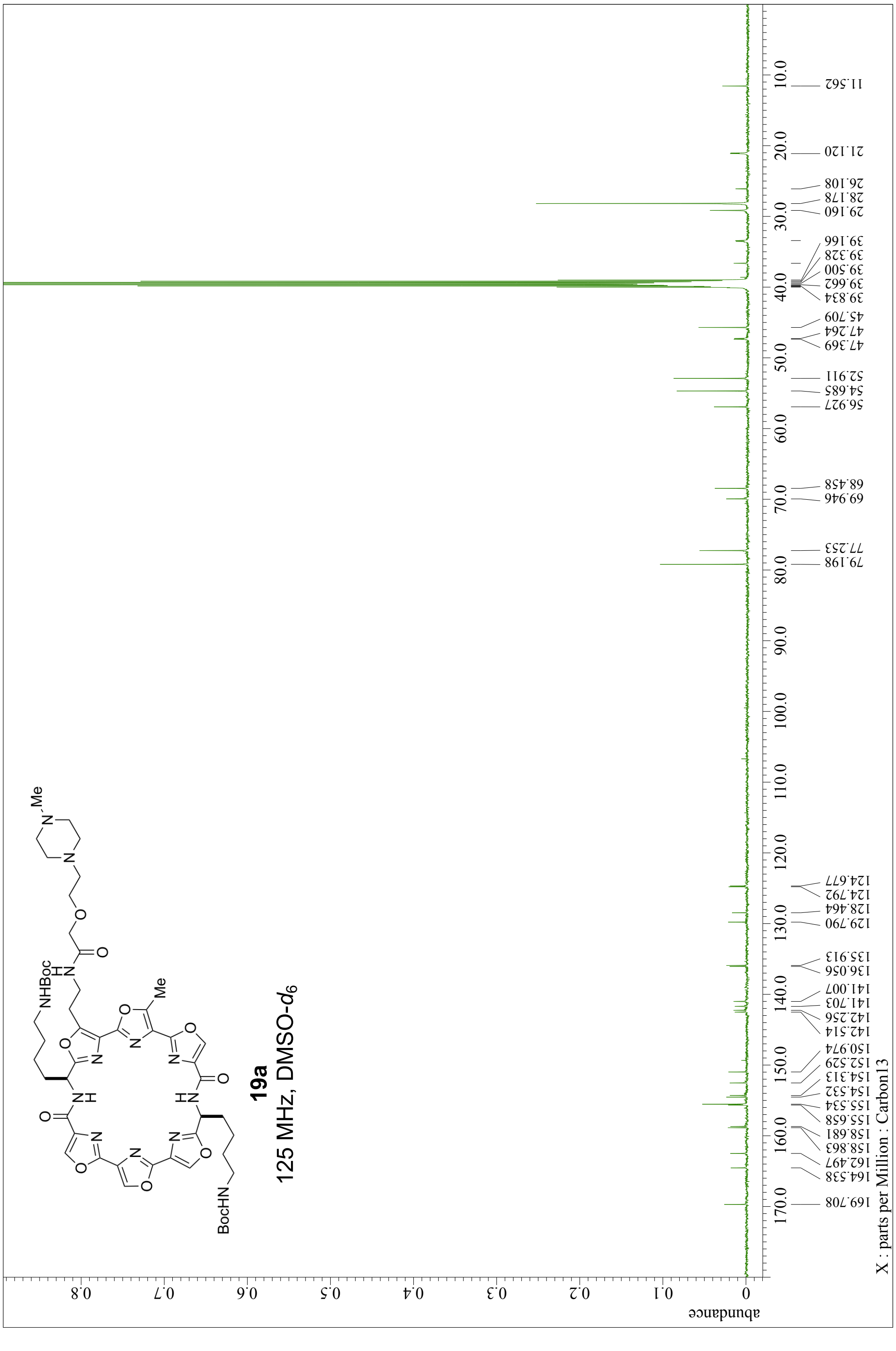
19a
125 MHz, DMSO-d_6
NHBoc
BocHN
Me
X : parts per Million : Carbon13
abundance
11.562
21.120
26.108
28.178
29.160
39.166
39.328
39.500
39.662
39.834
45.709
47.264
47.369
52.911
54.685
56.927
68.458
69.946
77.253
79.198
124.677
124.792
128.464
129.790
135.913
136.056
141.007
141.703
142.256
142.514
150.974
152.529
154.313
154.532
155.534
155.658
158.681
158.863
162.497
164.538
169.708

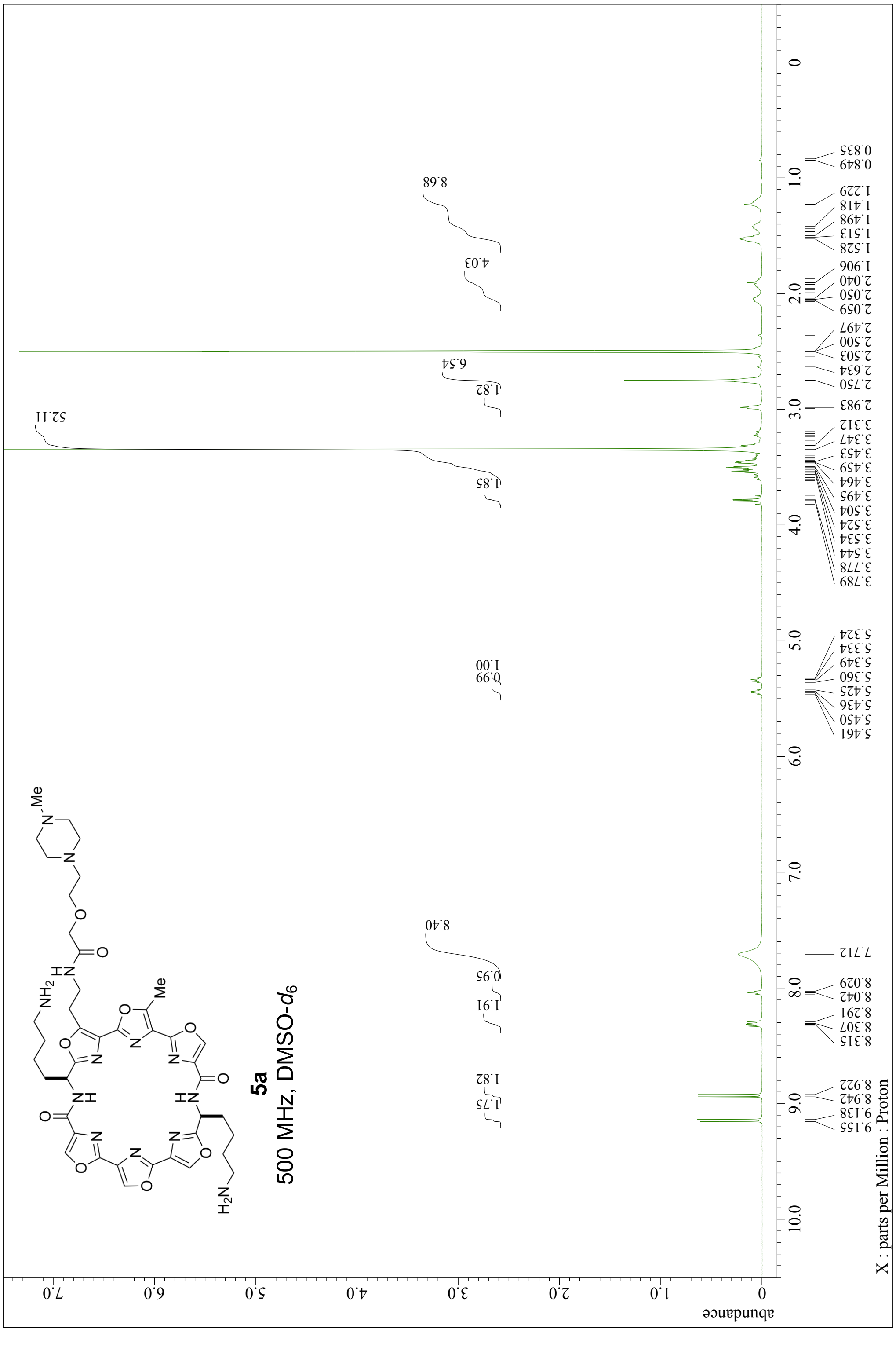

5a
500 MHz, DMSO-d_6
X : parts per Million : Proton
abundance
9.155
9.138
8.942
8.922
8.315
8.307
8.291
8.042
8.029
7.712
5.461
5.450
5.436
5.425
5.360
5.349
5.334
5.324
3.789
3.778
3.544
3.534
3.524
3.504
3.495
3.464
3.459
3.453
3.347
3.312
2.983
2.750
2.634
2.503
2.500
2.497
2.059
2.050
2.040
1.906
1.528
1.513
1.498
1.418
1.229
0.849
0.835

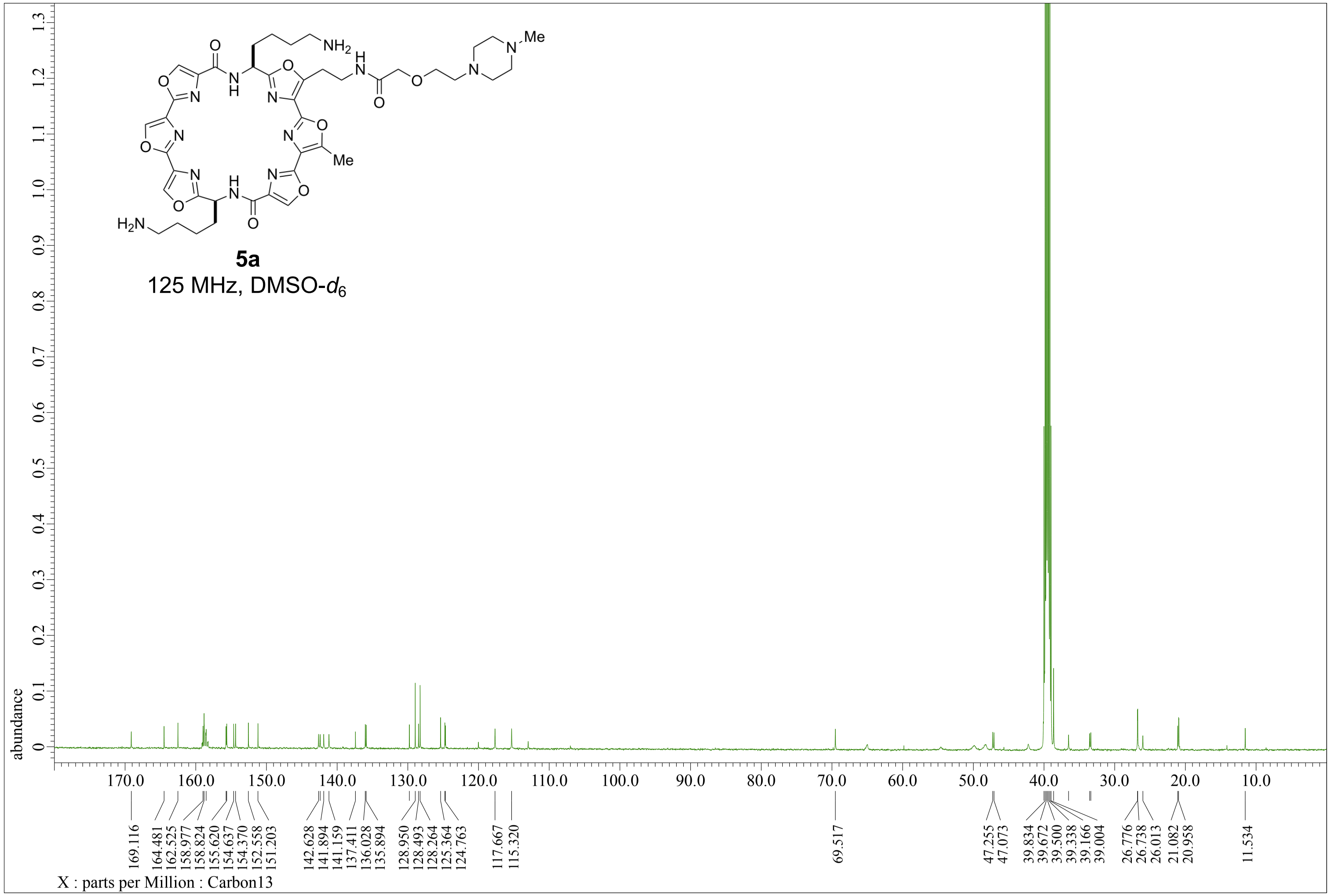
5a
125 MHz, DMSO-d6
abundance
X : parts per Million : Carbon13
169.116
164.481
162.525
158.977
158.824
155.620
154.637
154.370
152.558
151.203
142.628
141.894
141.159
137.411
136.028
135.894
128.950
128.493
128.264
125.364
124.763
117.667
115.320
69.517
47.255
47.073
39.834
39.672
39.500
39.338
39.166
39.004
26.776
26.738
26.013
21.082
20.958
11.534

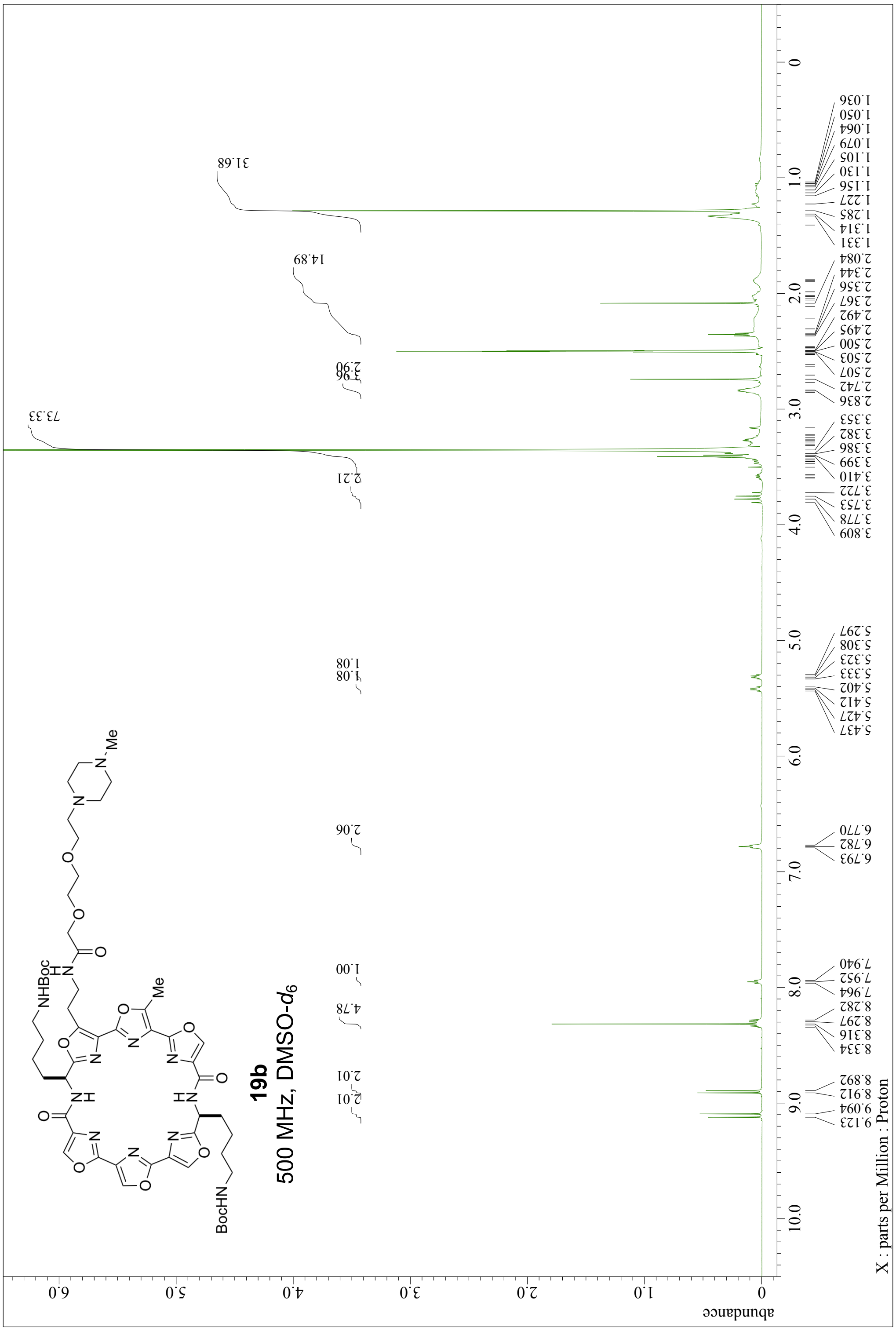
19b
500 MHz, DMSO-d_6
X : parts per Million : Proton
abundance
NHBoc
BocHN
Me

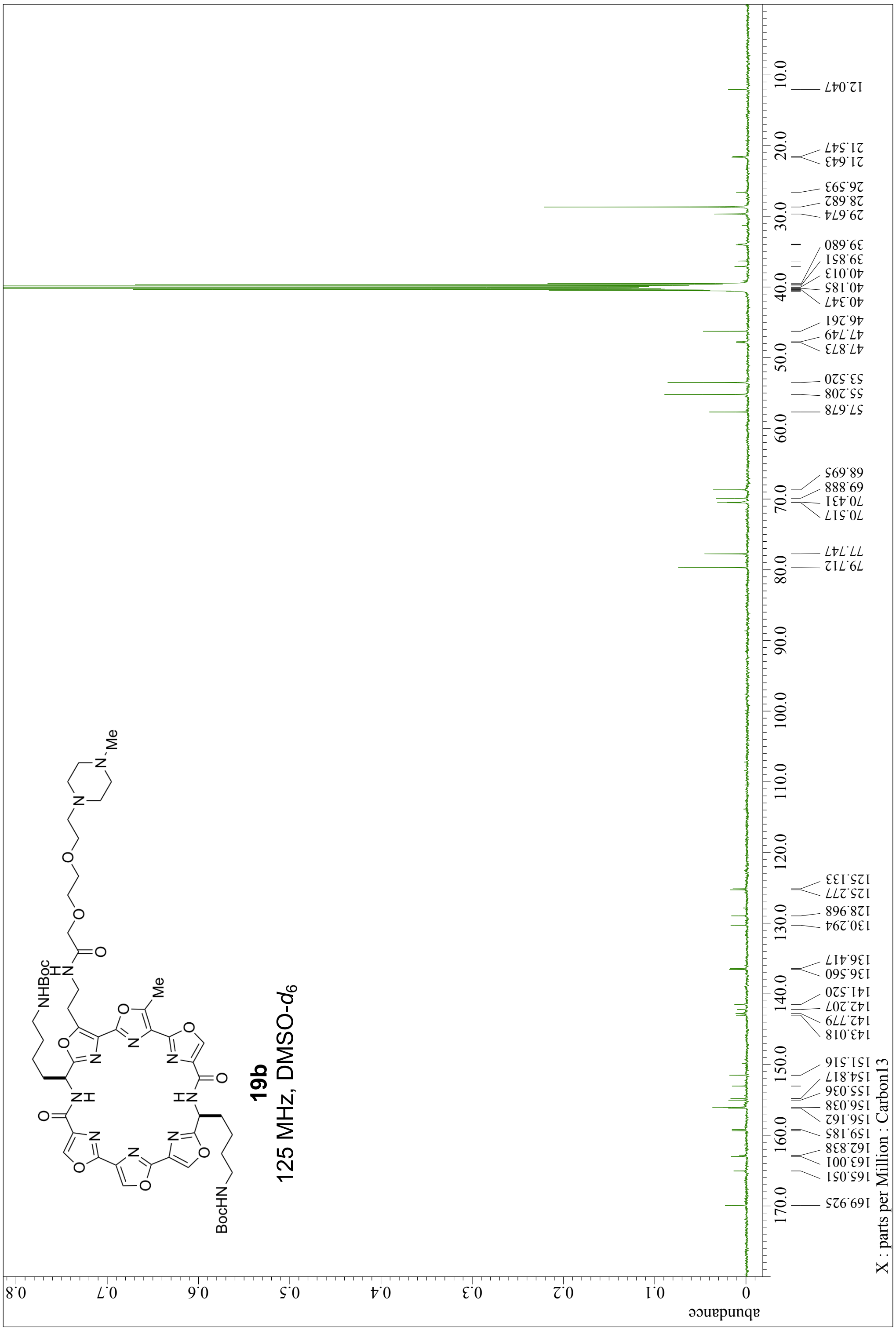
19b
125 MHz, DMSO-d_6
NHBoc
BocHN
Me
X : parts per Million : Carbon13
abundance
12.047
21.547
21.643
26.593
28.682
29.674
39.680
39.851
40.013
40.185
40.347
46.261
47.749
47.873
53.520
55.208
57.678
68.695
69.888
70.431
70.517
77.747
79.712
125.133
125.277
128.968
130.294
136.417
136.560
141.520
142.207
142.779
143.018
151.516
154.817
155.036
156.038
156.162
159.185
162.838
163.001
165.051
169.925

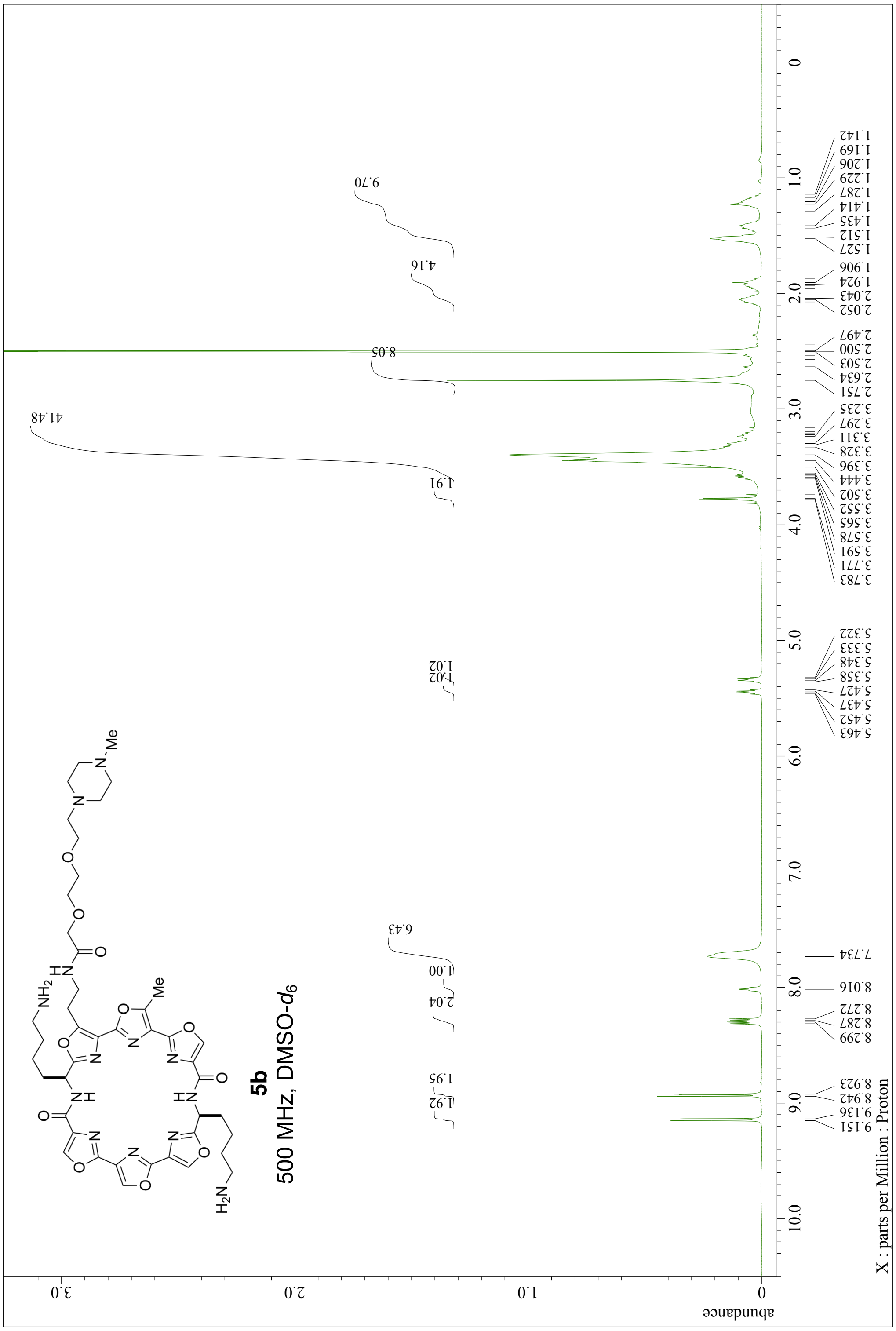
5b
500 MHz, DMSO-d_6
X : parts per Million : Proton
abundance

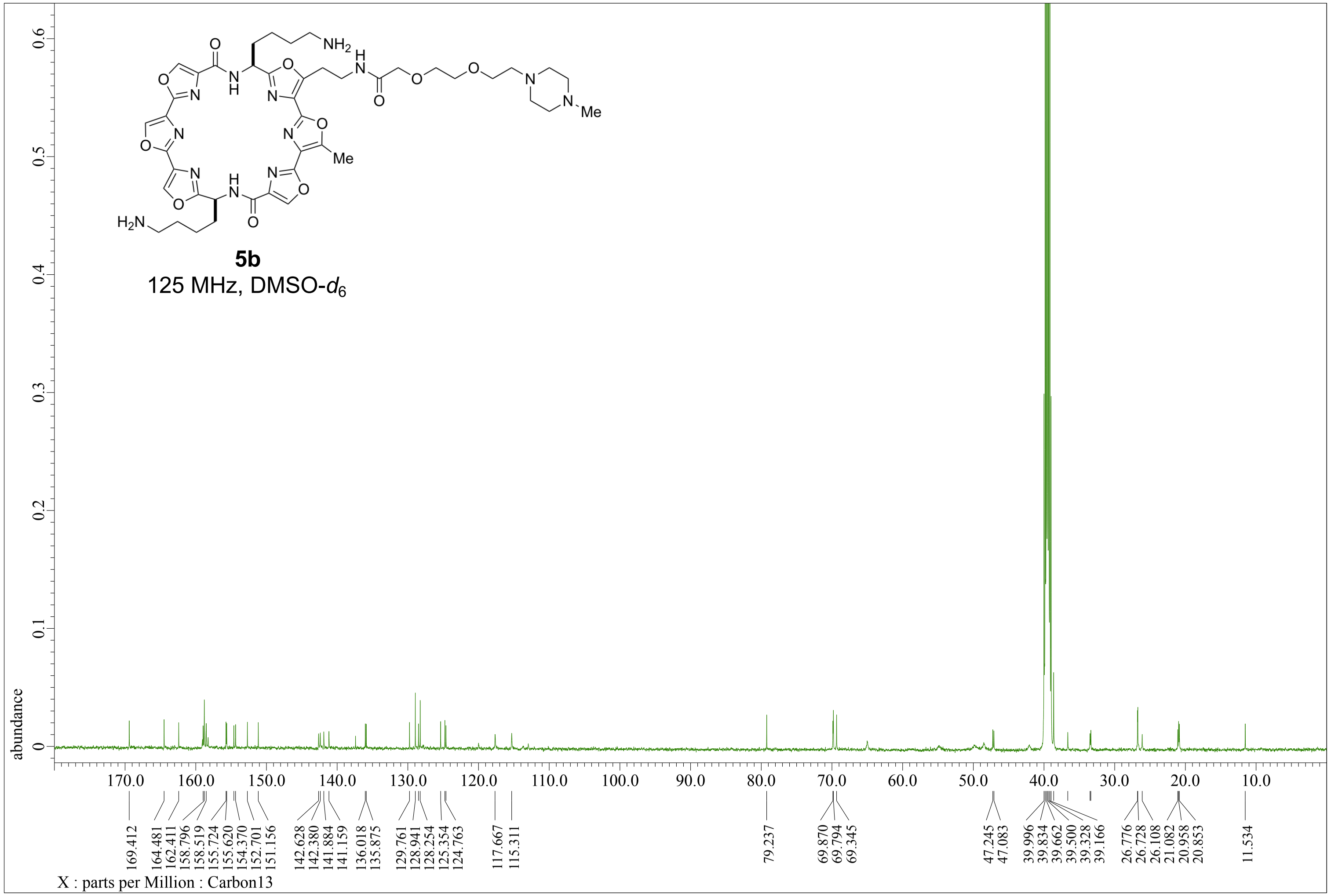

5b
125 MHz, DMSO-d_6
abundance
X : parts per Million : Carbon13
169.412
164.481
162.411
158.796
158.519
155.724
155.620
154.370
152.701
151.156
142.628
142.380
141.884
141.159
136.018
135.875
129.761
128.941
128.254
125.354
124.763
117.667
115.311
79.237
69.870
69.794
69.345
47.245
47.083
39.996
39.834
39.662
39.500
39.328
39.166
26.776
26.728
26.108
21.082
20.958
20.853
11.534
